# Liver Gene Therapy in Fabry Disease Mice With Low Doses of rAAV2/8 Expressing a Codon‐Optimized h *GLA* cDNA Results in Long‐Term Disease Correction

**DOI:** 10.1002/jimd.70188

**Published:** 2026-04-27

**Authors:** Himanshi Saxena, Rossana Domenis, Giulia Romano, Jessica Biasizzo, Martina Ferro, Dania Ferino, Antonio Vicidomini, Alessandra Iaconcig, Giulia Bortolussi, Lorena Zentilin, Andrea Dardis, Andrés F. Muro

**Affiliations:** ^1^ International Centre for Genetic Engineering and Biotechnology Trieste Italy; ^2^ Institute of Clinical Pathology, Department of Laboratory Medicine University Hospital of Udine Udine Italy; ^3^ Regional Coordinator Centre for Rare Diseases University Hospital of Udine Udine Italy

**Keywords:** AAV‐mediated liver gene therapy, alpha galactosidase A, Fabry mice, gene therapy, hot plate test, long‐term treatment

## Abstract

Fabry disease (FD) is an X‐linked lysosomal storage disorder caused by mutations in the *GLA* gene, which encodes for Alpha Galactosidase‐A (α‐Gal A). α‐Gal A deficiency leads to glycosphingolipid accumulation, like globotriaosylceramide (Gb3) and its deacylated form, globotriaosylsphingosine (lyso‐Gb3), resulting in systemic symptoms and reduced lifespan. Current treatments such as enzyme replacement therapy (ERT) and chaperone therapy are noncurative and have limitations. Gene therapy is an interesting alternative approach that may overcome most of these limitations, and different approaches are currently being tested. Here, we developed a gene therapy approach using rAAV2/8 vectors, delivered intravenously, that target the liver to produce and secrete functional α‐Gal A into circulation. This enzyme is then captured by organs expressing mannose‐6‐phosphate receptors, reducing glycosphingolipid accumulation in affected tissues. We generated a codon‐optimized *GLA* cDNA with enhanced translatability that was expressed under a strong liver‐specific promoter. In a dose escalation study in juvenile *Gla* knock‐out (ko) mice, the lowest dose (3.0E11vg/kg) resulted in 85%–95% clearance of lyso‐Gb3 in plasma and tissues, while doses of 3.0E12 vg/kg and higher showed 98%–100% clearance of the glycosphingolipid. All AAV doses were more effective than systemic α‐Gal A administration (ERT). Long‐term treatment showed normal levels of lyso‐Gb3 in plasma and tissues, and corrected neuropathic involvement, as shown in the hot plate test. This study provides a proof‐of‐concept showing that the tested liver‐specific gene therapy vector is capable of preventing disease progression in juvenile Fabry mice at relatively low doses and shows potential in treating both early‐ and late‐onset FD in patients.

## Graphical abstract

AAV‐mediated gene therapy targets the liver to produce and secrete into circulation functional α‐Gal A, which is taken up by tissues to reduce glycosphingolipid accumulation and prevent disease progression in juvenile Fabry mice, even at low AAV doses.

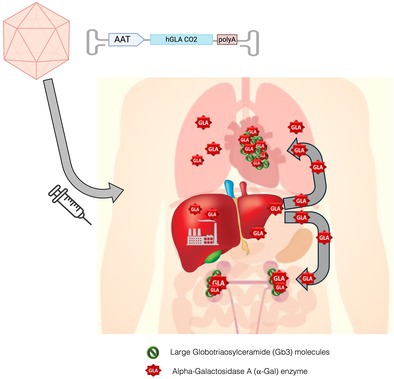

## 1 Introduction

Fabry disease (FD; OMIM: 301500) is an X‐linked lysosomal storage disorder caused by pathogenic variants in the *GLA* gene encoding α‐galactosidase (α‐Gal A). Enzyme deficiency leads to lysosomal accumulation of glycosphingolipids, primarily globotriaosylceramide (Gb3) and its deacylated form, globotriaosylsphingosine (lyso‐Gb3) [1, 2, 3]. Progressive substrate deposition results in cellular dysfunction and multi‐organ damage. Disease severity correlates with residual enzyme activity: individuals with 1%–3% activity develop the classical, early‐onset form that manifests during childhood or adolescence. Patients with 3%–30% of residual activity display the atypical or late‐onset FD, which manifests during adulthood [4, 5, 6].

FD has a reported frequency of 1 in 40 000 to 1 in 117 000 males [7, 8], but the accuracy of epidemiological data is compromised due to its pan‐ethnic nature [2]. Newborn screening predicts much higher incidence, that is, 1 in 1368 males in Taiwan [9], and 1 in 3100 to 1 in 4121 males in Italy, with an 11:1 ratio of late‐onset to classic cases [10, 11].

The current standard of care is enzyme replacement therapy (ERT) with recombinant α‐Gal A (agalsidase alfa and agalsidase beta) [12]. However, it presents important limitations: frequent infusions due to short half‐life [12, 13, 14, 15], high cost (~€200 k/year) [12, 16, 17], infusion reactions, and frequent development of anti‐drug antibodies, reducing therapeutic efficacy [18]. A second generation ERT has been recently approved (pegunigalidase alfa) is expected to increase half‐life and reduce immunogenicity [19, 20, 21]. Chaperone therapy (Migalastat/Galafold) is approved patients carrying amenable GLA variants [22]. Other approaches such as substrate reduction therapies are being tested [20].

Gene therapy has emerged as a promising long‐term alternative [23, 24]. Recombinant adeno‐associated vectors (rAAV) have demonstrated encouraging outcomes in both clinical and preclinical investigations of various inherited diseases, including Fabry disease [25, 26, 27, 28, 29, 30, 31, 32, 33]. Liver‐directed strategies benefit from the tolerogenic properties of this organ and reduced anti‐drug antibody formation [34, 35]. Preclinical FD studies demonstrated efficient hepatic α‐Gal A production and antigen‐specific tolerance [32, 33].

In this study, we evaluated a liver‐directed rAAV2/8 vector encoding human GLA cDNA under a liver‐specific promoter [36, 37, 38], in *Gla* knockout mice [39]. Our findings provide proof‐of‐concept for durable, low‐dose AAV‐mediated liver gene therapy in Fabry disease.

## 2 Materials and Methods

### 2.1 Codon‐Optimization of Human *GLA* cDNA

The human mRNA transcript 201 sequence (h*GLA* WT) was extracted from the NCBI database. Codon optimization was done using online tools applying different optimization algorithms (IDT Codon Optimization Tool, JCAT Java Codon Adaptation Tool, COOL Codon Optimization Online by the National University of Singapore). GC content was registered using ENDMEMO GC content calculator, CpG Islands were identified by EMBOSS CPG PLOT by EMBL‐EBI, and Cryptic Splice Sites were identified using Splice Site Prediction by Neural Network at Fruitfly.org. Further, the four codon‐optimized sequences obtained were manually edited to reduce splice site score and to avoid additional open reading frames in both strands and CpG Islands. A Kozak sequence was added at the 5′ end (5 GCCGCCACC 3′). All four codon‐optimized sequences along with the wild‐type sequences (h*GLA*_WT, h*GLA*_CO1, h*GLA*_CO2, h*GLA*_CO3, and h*GLA*_CO4) were synthesized by Genscript, cloned into the pUC57‐Kana plasmid, and transferred into the pSMD2 vector [38], containing the alpha‐1‐antitrypsin (hAAT), liver‐specific promoter, the apolipoprotein E (ApoE) enhancer, and AAV ITRs.

### 2.2 In Vitro Testing of rAAV Vectors

Huh‐7 cells [kindly provided by Alessandro Marcello, ICGEB, Trieste, Italy; [40]] were maintained in Dulbecco's Modified Eagle Medium (DMEM) with 10% of Foetal Bovine Serum (FBS). Transient transfection was performed using Lipofectamine 2000 reagent following the manufacturer's guidelines (Lipofectamine 2000 Transfection Reagent; Cat# 11668019; Invitrogen). Cells were incubated at 37°C for 48 h.

### 2.3 Animals

All animals were housed and handled according to the institutional guidelines and the Italian Ministry of Health in the ICGEB bio‐experimental facility in Trieste (Project Authorization N. 365/2021‐PR), following European Union (EU) Directive 2010/63/EU for animal experimentation and ARRIVE guidelines. C57Bl/6 WT and *Gla* knock‐out (ko) mice were maintained in a temperature‐controlled environment with 12/12 h of light/dark cycles and received a standard chow diet and water ad libitum. *Gla* ko mice or B6;129‐GLA^tm1Kul/J^ (Strain #003535) were purchased from Jackson laboratory and were housed in the ICGEB bio‐experimental facility. Only male mice were used for experimentation.

### 2.4 Animal Procedures

AAV viral vector treatment was done intravenously via the retro‐orbital route, dosed as AAV viral genomes per animal weight (vg/kg) in isoflurane anesthetized animals as previously described [41].

For ERT treatment, 3 months‐old GLA ko male animals were treated with 1 mg/kg agalsidase alfa (Replagal Takeda, ERT) every week for 2 months. Blood samples were taken every 2–4 weeks, and mice were sacrificed at P150, 2 months after ERT first dosing.

To assess pain sensitivity, a hot plate assay was conducted on age‐matched male mice (330 days old) as previously described with minor modifications [32]. Following a 20‐min acclimation period, each mouse was placed on a 50°C heated metal surface enclosed by an 18 cm diameter cartoon wall to prevent escape. A timer was started, and the latency (in seconds) to the first sign of nociception (hind paw licking, shaking, flicking, jumping, or vocalizing) was recorded. A 30‐s cut‐off was implemented to avoid tissue damage.

### 2.5 Viral Genomes Determination

Genomic DNA was extracted from pulverized liver using the Wizard SV genomic DNA purification system (Promega) following the manufacturer's guidelines. Vector genomes in liver were quantified by real‐time PCR using the iQ SYBER green supermix (Bio‐Rad), as previously described [37].

### 2.6 Lyso‐Gb3 Quantification

Lyso‐Gb3 level was evaluated in murine plasma and tissues (liver, kidney, and heart) of treated and untreated animals. For tissues, 20 mg of powdered tissue was homogenized using a mechanical homogenizer (IKA ULTRA‐TURRAX T25) with 350 μL of water. Tissue and plasma lyso‐Gb3 concentrations were determined by liquid chromatography‐mass spectrometry (LCMS) at Centro Servizi di Laboratorio (CSL) in Hospital Santa Maria della Misericordia in Udine. Homogenized samples or plasma were mixed with precipitation solution (10 mL internal standard solution (1 ng/mL‐10 ng/mL), 10 mL acetone, 2.22 mL water) containing internal standard (1 ng/mL Lyso GlcCer d5—Lyso‐SM d9; 10 ng/mL Lyso Gb3 d7 in Methanol). The mix was vortexed and incubated on an orbital shaker for 30 min and then centrifuged at 16 000 rcf for 10 min. The clear supernatant was collected and dried under a nitrogen stream. The dried content was reconstituted with 100 μL mobile phase (55% FMA: water + 0.1% HCOOH, 45% FMB: CH3CN + 0.1% HCOOH) and centrifuged at 16 000 rcf for 5 min. One hundred microliters of supernatant was used for analysis by injecting 2 μL in Citrine Sciex 6500QT with Poroshell 120 EC‐C8 2.7 μm and 3.0 × 50 mm columns. The analysis was done against a calibration curve prepared by spiking the pure standards in water: methanol 50:50 solution (range 0.1–200 ng/mL for LysoGb3, Lyso GlcCer, and range 1–2000 ng/mL for Lyso SM/509).

In some figures, the absolute values of lysoGb3 accumulation in plasma (ng/ml) and tissues (ng/mg) were converted to percentages for ease of understanding. In the case of plasma, the mean of lyso‐Gb3 accumulation in untreated hemizygous males (*n* = 5) at P150 was considered as 100%. Whereas, in the case of tissues, the mean of lyso‐Gb3 accumulation in the specific organ (liver/kidney/heart) of untreated hemizygous males (*n* = 5) was considered as 100% accumulation. All the treated and untreated wild‐type substrate levels were re‐calculated as percentages based on this criterion.

### 2.7 Statistics

Statistical analyses were performed for all the assays using GraphPad Prism 10.3.0. Data in the graphs were plotted as mean with the corresponding standard deviation (SD). Statistical analysis of data containing different time points was done using a mixed‐effect model (REML). The comparison of groups was done using one‐way or two‐way ANOVA. A *p*‐value below 0.05 was considered significant (*p < 0.05, **p < 0.01, ***p < 0.001).

## 3 Results

### 3.1 Generation and Evaluation of Codon‐Optimized Human *GLA* cDNA Constructs In Vitro

**FIGURE 1:**
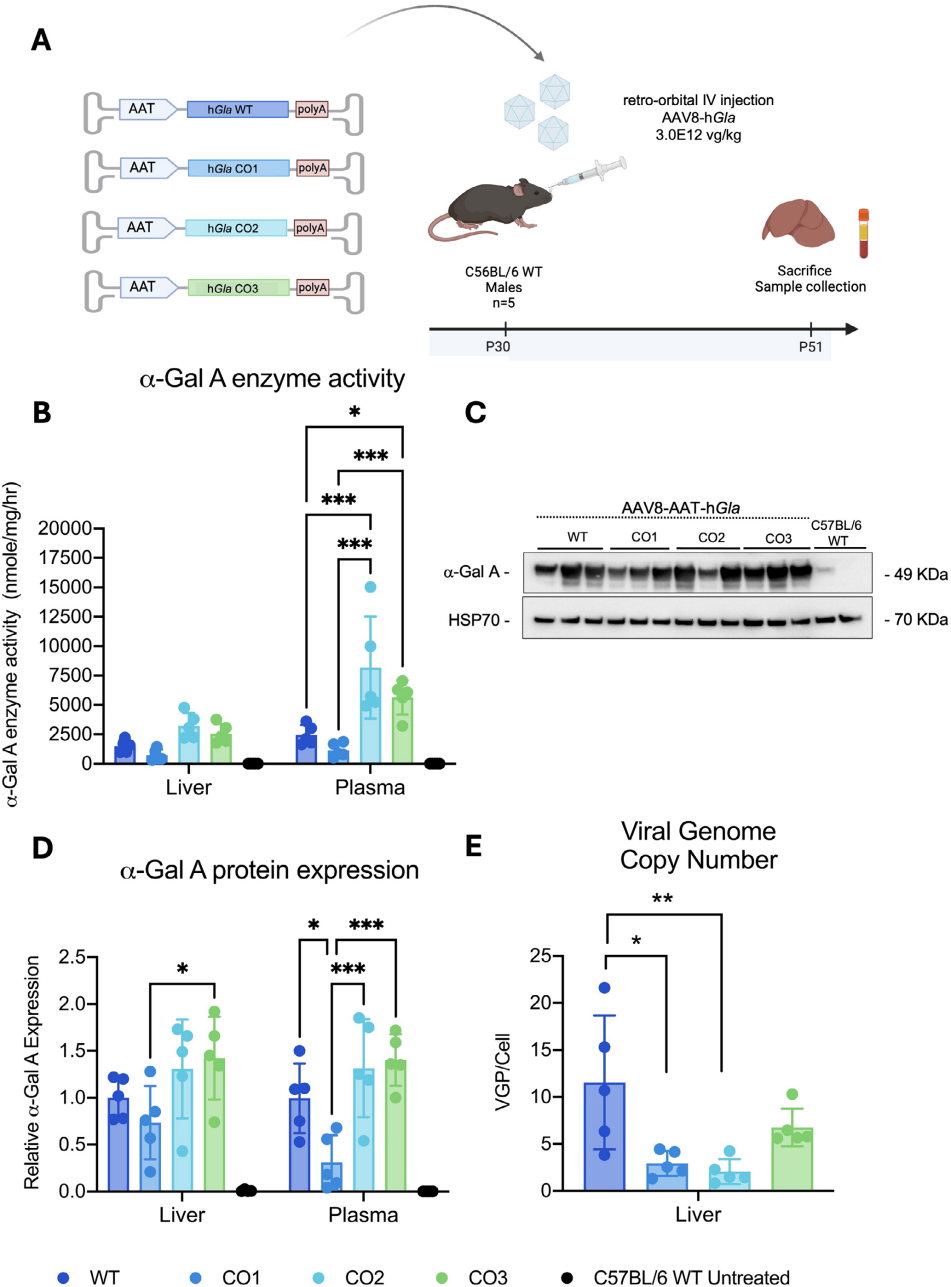
Assessment of the codon‐optimized sequences in vivo in WT mice. (A) Experimental design for the assessment of the codon‐optimized sequences in vivo in C57BL/6 WT mice. AAV8 viral stocks were used to transduce C57Bl/6 WT mice (*n* = 5) at 3.0E12vg/kg intravenously (retro‐orbital) at P30. The human alpha‐1 antitrypsin promoter (hAAT) and apolipoprotein E enhancer elements (ApoE) provide liver‐specific gene expression. The experiment was terminated after 3 weeks (P51), harvesting the liver and collecting blood for plasma analysis; (B) The bar graph shows the α‐Gal A enzyme activity in liver protein extracts of AAV‐transduced mice; (C) Western blot of liver protein extracts using an anti‐α‐Gal A antibody. HSP70 was used as housekeeping gene to normalize protein expression; (D) Quantification of liver and plasma western blots; (E) The viral genome copies was quantified in genomic DNA samples extracted from liver homogenates. *p < 0.05, **p < 0.01, ***p < 0.001.

We generated a series of codon‐optimized *GLA* cDNA variants by applying different codon optimization algorithms, followed by further manual optimizations for GC content, CpG islands, cryptic splice sites, and alternative ORF in the coding and non‐coding strands (Figure S1A–C; Table S1). These variants were cloned into a liver‐specific AAV pSMD2 vector and tested for α‐Gal A expression and enzyme activity in vitro and in vivo. Four codon‐optimized constructs were selected for in vitro testing: h*GLA*_CO1, h*GLA*_CO2, h*GLA*_CO3, and hGLA_CO4 with 76.52%, 78.11%, 80.23%, and 80.76% sequence identity with the h*GLA* WT cDNA sequence, respectively (Table S1 and Figures S2 and S3). The pSMD2 plasmid contains liver‐specific promoter and enhancer sequences (alpha‐1 antitrypsin, α1‐hAAT, and apolipoprotein E enhancer, ApoE) (Figure 1A and Figure S1A) [38], providing hepatocyte‐specific expression of the transgene [36, 37]. We first compared α‐Gal A protein expression and enzymatic activity between the CO variants and the WT *GLA* cDNA by transiently transfecting Huh‐7 liver cells (Figure S1D–F). The variants CO1, CO2, and CO3 showed about two‐fold increased α‐Gal A protein expression and enzyme activity in the cell extracts compared to the WT *GLA* cDNA. The enzyme activity and protein levels of these variants in the supernatant were 4‐ and 6‐fold increased, respectively, compared to the WT enzyme, suggesting improved secretion. The CO4 cDNA showed the lowest activity and was not further tested.

### 3.2 Evaluation of the Codon‐Optimized Human *GLA* cDNA Constructs In Vivo in WT Mice

Next, we tested the pSMD2‐CO1, ‐CO2, and ‐CO3 constructs in vivo in WT animals. We prepared AAV8 stocks and dosed C57Bl/6 juvenile P30 male mice with 3.0E12 vg/kg of the different CO and WT versions and collected plasma and liver 3 weeks after the treatment (Figure 1A). Enzyme activity in the liver and in plasma showed increased levels in animals dosed with the CO2 and CO3 *GLA* cDNA versions, while those treated with CO1 *GLA* cDNA showed lower activity levels compared to mice treated with the WT construct (Figure 1B). These results were confirmed by Western blot (Figure 1C,D; Figures S4 and S5). Viral genome copies (VGC) were lower in the livers of animals treated with the CO versions compared with the group treated with the WT cDNA, indicating that these differences were probably higher (Figure 1E).

Thus, considering the in vitro and in vivo results, we selected the CO2 *GLA* cDNA for the next set of experiments.

### 3.3 Testing the Codon‐Optimized Constructs in *Gla* Knock‐Out Mice

We then tested the effective therapeutic activity of the CO2 *GLA* cDNA in *Gla* ko male mice (Fabry mice). These animals (Jackson Laboratory, Main, USA; Cat no. 003535) present a complete loss of *Gla* gene expression [39], accumulate Gb3, similar to Fabry patients, but their disease progression is relatively slow and key clinical features are absent resembling late‐onset phenotype [42]. As expected, we observed no α‐Gal A enzyme activity in liver, kidney, heart, and plasma (Figure S6A–D), and accumulation of lyso‐Gb3 in plasma, liver, and kidney (Figure S5E,F).

**FIGURE 2:**
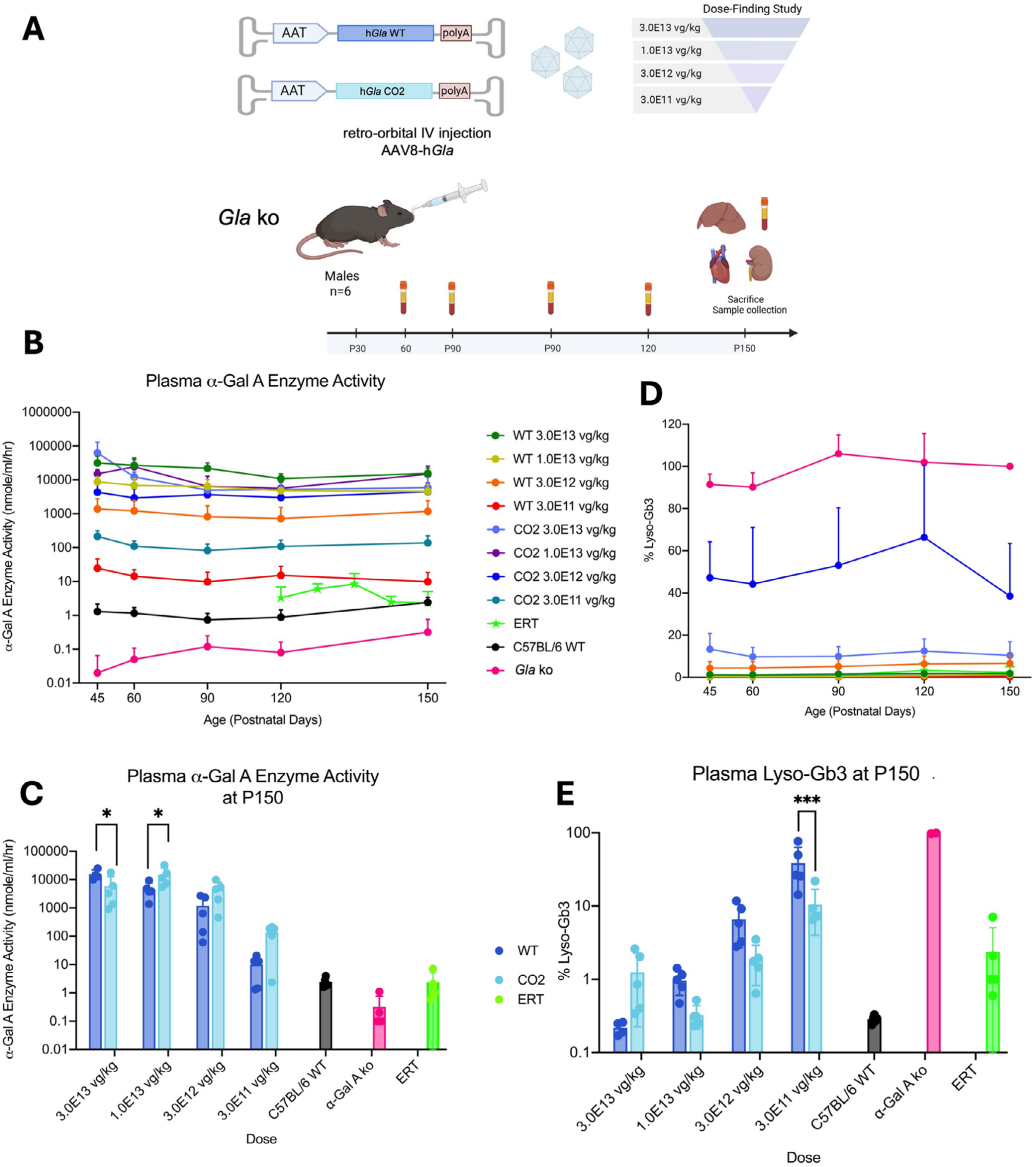
Assessment of the codon‐optimized cDNA variants in vivo in Fabry mice treated with AAV‐based gene therapy. (A) Experimental scheme. Hemizygous male mice (B6;129‐GLA^tm1Kul/J^; *n* = 5 per group) were treated with either AAV8‐pSMD2_h*GLA* WT or AAV8‐pSMD2_h*GLA* CO2 viral vectors at the dose of 3.0E13 vg/kg, 1.0E13 vg/kg, 3.0E12 vg/kg and, 3.0E11 vg/kg. Animals were injected retro‐orbitally at P30 and sacrificed at 5 months of age. Blood was collected at intermediate time points and at termination along with harvesting the liver, kidneys, and heart for analysis. Age‐matched untreated hemizygous males (B6;129‐GLA^tm1Kul/J^) and wild‐type (B6;129sf2/J) were used as controls; (B) α‐Gal A enzyme activity was measured in blood plasma collected at different time points at P45, P60, P90, P120, and P150 for all treated and untreated animals. The graph shows the mean of the activity (nmoles/ml/h) at each time point; (C) Bar graph representation of enzyme activity at P150. Each dot represents a single animal; (D) Lyso‐Gb3 levels were measured in blood plasma collected at different time points during the experiment. The lyso‐Gb3 levels is indicated as a percentage, considering the mean lyso‐Gb3 levels in untreated hemizygous mice as 100%. (E) The bar graphs represent lyso‐Gb3 at P150. Each dot represents a single animal. *p < 0.05, ***p < 0.001.

We performed a side‐by‐side comparison between the CO2 and the WT *GLA* cDNA versions by dosing different amounts of AAV8 to Fabry mice (Figure 2A). Aiming at mimicking the treatment of juvenile patients affected by Fabry Disease, we injected P30 mice (retro‐orbital injection) with AAV8 doses ranging from 3.0E11 to 3.0E13 vg/kg. As a control, 3 months old Fabry KO animals were treated with enzyme replacement therapy (ERT, 1 mg/kg agalsidase alfa, Replagal, Takeda) every week for 2 months. It is important to note that the ERT dose used here is five‐fold higher than the one normally administered to patients (1.0 and 0.2 mg/kg, respectively), since it was previously demonstrated that a higher dose was required to obtain a complete or almost complete reduction of Gb3 and Lyso‐Gb3 accumulation in Fabry mice [43, 44]. Blood samples were taken every 2–4 weeks, and mice were sacrificed at P150, 4 months after AAV dosing, and 2 months after ERT first dosing. The weight curve was not affected by the treatment (Figure S7A,B). The ERT resulted in increased enzyme activity in plasma reaching the a‐Gal A activity levels of untreated wild‐type animals (Figure 2B,C). For the duration of the experiment and for all tested doses and GLA constructs, α‐Gal A enzyme activity in plasma was higher than the one observed in both WT mice and mutant mice treated with ERT (Figure 2B,C). In particular, the administration of the CO2 vector (AAV doses of 1.0E13, 3.0E12, and 3.0E11 vg/kg) resulted in an enzyme activity increase at P150 of ~6090‐, 1844, and 57‐fold vs. untreated WT mice, and 3.2‐, 3.8‐, and 13.9‐fold increase of enzyme activity, respectively, compared to mice treated with the WT *GLA* cDNA (Figure 2B,C). Plasma lyso‐Gb3 level was reduced in all treated animals, compared to untreated Fabry mice, showing a clear dose–response effect (Figure 2D,E; Table S2). At P150, the lyso‐Gb3 levels in plasma of AAV‐treated Fabry mice were reduced by 99.7%, 98.1%, and 89.5%, for the AAV doses of 1.0E13, 3.0E12, and 3.0E11 vg/kg, respectively, compared to untreated Fabry mice (Figure 2D,E). In line with the results of the enzyme activity, lyso‐Gb3 clearance was more pronounced in the animals treated with the CO2 *GLA* cDNA compared to those treated with the WT *GLA* cDNA (3.0‐, 3.5‐, and 3.7‐fold increased reduction of CO2 cDNA vs. WT cDNA, for the AAV doses of 1.0E13, 3.0E12, and 3.0E11 vg/kg, respectively; Figure 2D,E; Table S2). Unexpectedly, we did not observe a dose–response effect at the late timepoints with the dose of 3.0E13 vg/kg CO2 cDNA, which showed lower enzyme activity and lower clearance of lyso‐Gb3 in the plasma, compared to the animals treated with the same vector at a lower dose (1.0E13 vg/kg). However, we observed higher enzyme activity in the early timepoints which was gradually lost, while the dose–response effect was present in the animals treated with the higher dose of the WT cDNA (3.0E13 vg/kg). We hypothesize that these differences may be related to the activation of endoplasmic reticulum (ER) stress by the excessive over‐production of a‐Gal A in the hepatocytes transduced by the CO2 *GLA* cDNA, but not in those transduced by the WT *GLA* cDNA.

At sacrifice, blood, liver, kidney and heart were collected and VGC, α‐Gal A levels and enzyme activity, and lyso‐Gb3 accumulation were determined. The Western blot analysis of α‐Gal A protein in liver protein extracts showed a dose–response increase in the animals treated with AAV vector (Figure S7C,D), with higher α‐Gal A expression levels in the animals treated with the CO2 *GLA* cDNA (doses 1.0E13–3.0E11 vg/kg), compared to animals treated with the same doses of the WT cDNA construct, although these differences were not statistically significant.

**FIGURE 3:**
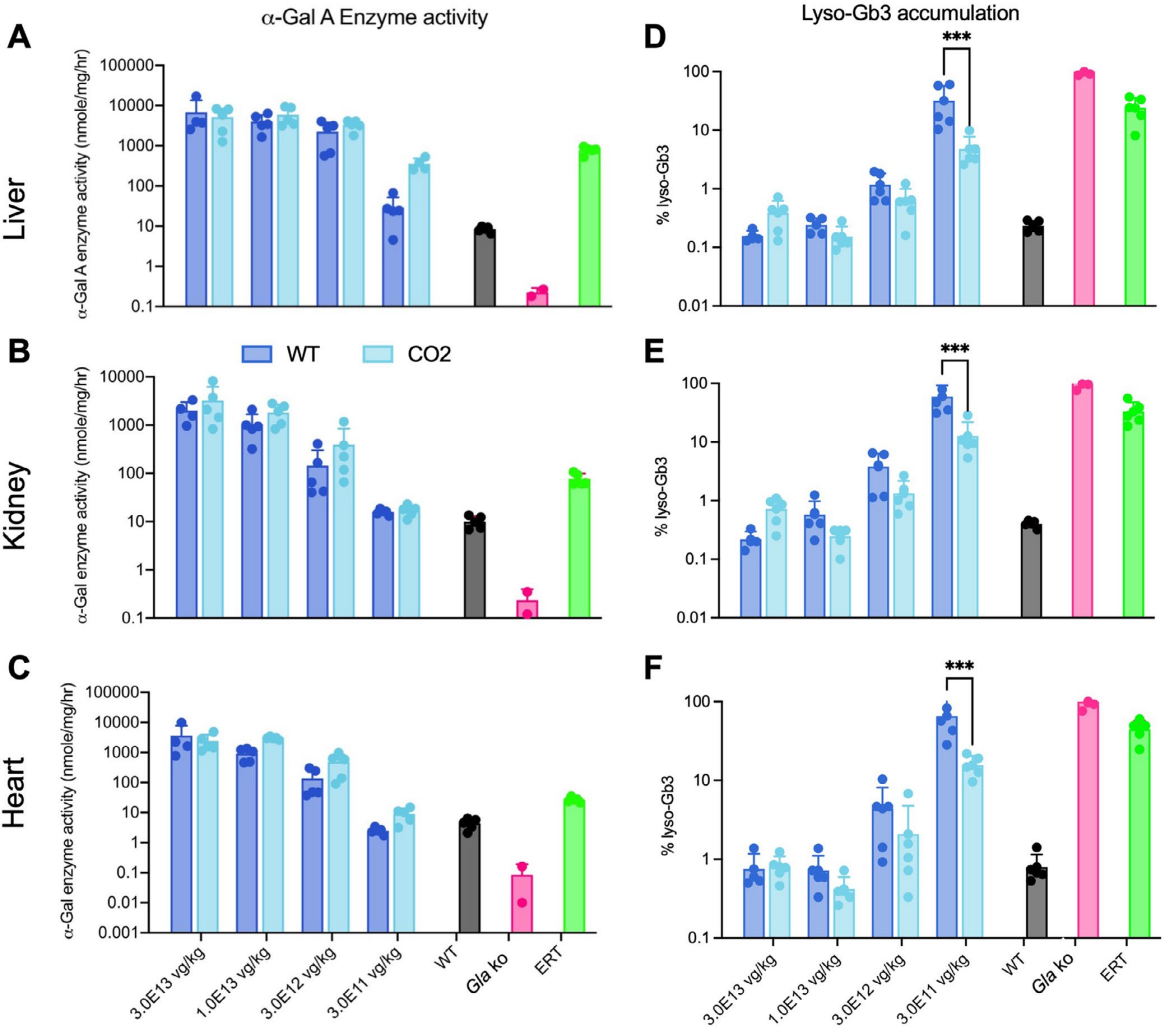
Evaluation of enzyme activity and lyso‐Gb3 accumulation in tissues of Fabry KO mice treated with AAV‐based gene therapy. α‐Gal A enzyme activity was measured in protein extracts from (A) liver (B) Kidney, and (C) heart (nmoles/mg/h) of the experiment shown in Figure 2. The bars represent the mean of α‐Gal A enzyme activity after 4 months of treatment. The lyso‐Gb3 accumulation was determined in (D) liver, (E) kidney, and (F) heart tissue homogenates. The bars represent the mean percentage lyso‐Gb3 levels after 4 months of treatment. Untreated WT and Fabry mice, as well as Fabry mice treated since M3 with ERT are shown. Each dot represents a single animal. ***p < 0.001.

In the ERT‐treated animals, we observed a supraphysiological increase of α‐Gal A activity in the liver, kidneys, and heart (Figure 3A–C), leading to a 76% reduction in substrate accumulation in the liver, 67% in the kidneys, and 56% in the heart, compared to the values of untreated Fabry mice (Figure 3D–F). A dose–response effect on α‐Gal A enzyme activity was also observed in the tissues of the AAV‐treated animals, with the CO2 GLA version resulting in higher enzyme activity levels compared to the WT construct (Figure 3A–C). For all tissues and AAV doses, the levels of α‐Gal A enzyme activity were equal to or higher than those of WT untreated controls. When compared to ERT‐treated mice, only in the groups treated with the lower AAV dose were the α‐Gal A enzyme activity levels lower than those of the ERT‐treated animals. The doses of 3.0E13, 1.0E13, and 3.0E12 vg/kg cleared lyso‐Gb3 to WT levels in the tested tissues (Figure 3D–F). For the dose of 3.0E11 vg/kg, the CO2 cDNA version was significantly more efficient than the WT version in clearing lyso‐Gb3 from tissues (95% vs. 68% in liver; 87% vs. 40% in kidney; and 84% vs. 34% in heart, respectively; Figure 3D–F).

Assessment of VGC in the liver showed similar levels between the CO2 and the WT AAV vectors for each of the different doses (Figure S7E).

### 3.4 *GLA* ‐AAV‐Gene Therapy Treatment in Fabry Mice Results in Long‐Term Correction of Lyso‐Gb3 Accumulation and Normal Performance in the Hot Plate Test

**FIGURE 4:**
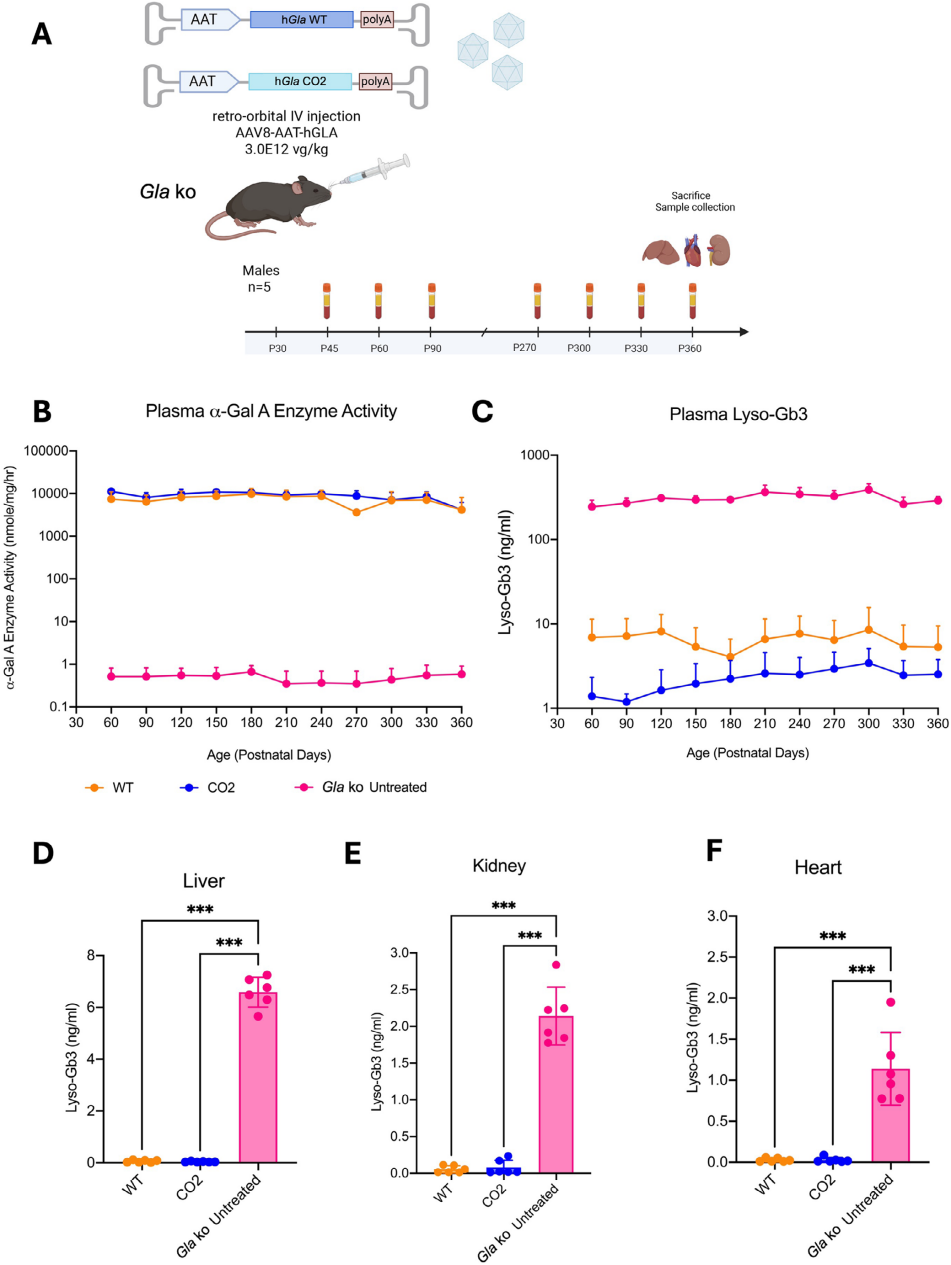
Long‐term evaluation of FD phenotype in plasma and tissues of Fabry KO mice treated with AAV‐based gene therapy. (A) Experimental scheme. Juvenile (P30) Fabry KO mice were treated with AAV8‐pSMD2_h*GLA* WT or AAV8‐pSMD2_h*GLA* CO2 viral vectors at the dose of 3.0E12 vg/kg. Blood was collected at intermediate time points and at termination along with harvesting the liver, kidneys, and heart for analysis. Age‐matched untreated hemizygous males (*GLA* ko: B6;129‐GLA^tm1Kul/J^) and wild‐type (B6;129sf2/J) were used as controls; (B, C) α‐Gal A enzyme activity (B) and lyso‐Gb3 accumulation (C) were measured in plasma. ***, *p* ≤ 0.001, RM‐ANOVA; (D–F) Lyso‐Gb3 accumulation was measured in tissue homogenates of (D) liver, (E) heart and (F) kidney after 11 months of treatment. The bars represent the mean percentage lyso‐Gb3 levels. ***, *p* ≤ 0.001, One‐Way ANOVA, Bonferroni Post hoc tests.

To assess the long‐term efficacy of the approach, we dosed a group of animals at P30 (3.0E12 vg/kg of CO2 or WT AAV vectors) and monitored the animals up to 12 months of age (Figure 4A). Blood samples were taken monthly, and enzyme activity and lyso‐Gb3 levels were determined.

α‐Gal A activity and lyso‐Gb3 showed stable levels in plasma during the whole study (Figure 4B,C). The plasma of mice dosed with the CO2 *GLA* cDNA version presented a minor, non‐statistically significant increase in enzyme activity compared to the group treated with the WT cDNA version (Figure 4B and Table S3). Quantification of plasma lyso‐Gb3 levels was significantly lower in mice treated with the CO2 *GLA* cDNA compared to the WT cDNA (*p* ≤ 0.001; Figure 4C), confirming the higher therapeutic efficacy of the AAV vector expressing the codon‐optimized *GLA* cDNA. Quantification of lyso‐Gb3 in target tissues showed complete clearance in both groups of animals (Figure 4D–F), confirming the results of the shorter‐term experiment (Figure 3D–F) and the durability of the treatment.

**FIGURE 5:**
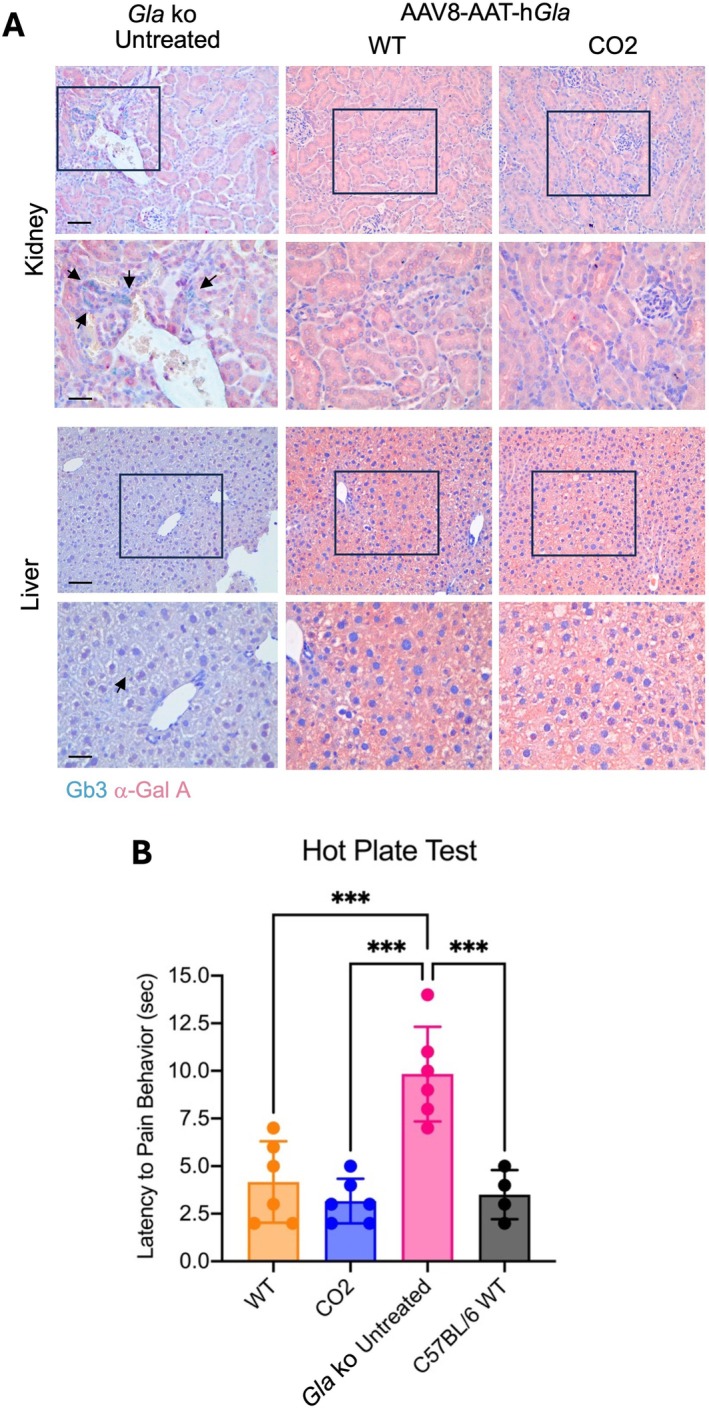
Histological and functional evaluation of Fabry KO mice treated long‐term with AAV‐based gene therapy. (A) Tissue sections of untreated controls and gene therapy‐treated mice were stained for lyso‐Gb3 accumulation. Kidney and liver sections are shown. The scale bar corresponds to 100 and 50 μm (kidney and liver, top panels, and kidney and liver, bottom panels, respectively). (B) Hot plate test. Treated and untreated control mice were challenged with the hot‐plate test and latency to pain was recorded. Each dot indicates a single animal. *** *p* < 0.001.

The histological analysis of tissue sections of liver and kidney showed the presence of Gb3 accumulation in the kidney and liver of untreated Fabry mice (emerald green, black arrows, Figure 5A), which were not present in the animals treated with either the WT or the CO2 GLA cDNA versions. These results are in complete agreement with the previous data, which showed normal levels of lyso‐Gb3 in tissues in animals of 5 months of age after the treatment at P30 with 3.0E12 vg/kg of the AAV vector (Figure 2D,E).

To evaluate the functional efficacy of the therapy we have tested the recovery of neurological symptoms in the treated animals. Thus, since Fabry mice exhibit hyposensitivity to heat [27, 45, 46], we challenged AAV‐treated and control animals with the hot plate test. We observed that the latency of untreated Fabry mice was two‐ to three‐fold higher than for WT and heterozygous mice. On the contrary, Fabry mice treated with AAV vectors (both the CO and WT cDNA groups) behaved as WT untreated animals (Figure 5B), indicating that the treatment of presymptomatic Fabry mice can prevent the decline in peripheral sensory function. These results strongly indicate that AAV8‐mediated liver gene transfer of the *GLA* cDNA into Fabry mice was able to correct not only the biochemical aberration but also a measurable functional deficit.

## 4 Discussion

Current treatments for FD rely on the administration of ERT or chaperone therapy. However, these therapeutic approaches present limitations, supporting the development of more effective therapeutic strategies. In vivo gene therapy, based on the direct injection of viral vectors, allows sustained expression of the α‐Gal A enzyme, offering several advantages over ERT. Liver gene therapy appears as a potential strategy to achieve a long‐term therapeutic effect with a single systemic application, with enzyme production occurring in a 24/7 fashion. On the contrary, ERT necessitates lifelong 2‐ to 4‐h biweekly infusions that, associated with the short half‐life of the recombinant enzyme in circulation [28], results in a pharmacokinetic profile presenting high levels immediately after infusion, rapidly decreasing to very low levels after a few days.

The liver is an excellent target for gene therapy due to its easy accessibility, its unique dual blood supply, and its tolerogenic properties. In fact, liver expression of a transgene driven by AAV vectors is associated with induction of robust peripheral tolerance to the transgene product with reduction in anti‐drug antibodies induction [33, 35]. Clinical application of AAV‐based vectors for liver‐targeted in vivo gene therapy has rapidly increased over the last decade, largely because of the good safety profile of these vectors and the success of recent clinical trials [26, 47, 48]. Importantly, the therapeutic effect of AAV‐mediated liver gene therapy appears to be sustained long‐term, as efficacy of the therapeutic gene has been documented for more than 8 years in hemophilia clinical trials [49], and it holds promises, at least for the treatment of some conditions, for once‐in‐a‐life‐time treatment [50]. However, physiological hepatocyte duplication and conditions influencing hepatocyte turnover may impact the long‐term efficacy of the therapy [51]. Published data established that human hepatocytes divide at an estimated rate of 17%–19% per year [52] and the lifespan of mouse hepatocytes was estimated in ~200 days [53]. This suggests that re‐administration of the therapeutic vector in a second moment may be necessary, but the induction of the humoral immune response against the AAV capsid, resulting in persistent neutralizing antibody titers, precludes repeated vector administration [29, 47, 54]. Several strategies are being tested to allow re‐administration of the AAV vector, ranging from the use of immunosuppressors to removal of the neutralizing antibodies by plasmapheresis or by the use of IgG‐cleaving endopeptidases [55, 56, 57, 58, 59, 60, 61, 62].

Here, we developed a codon‐optimized GLA cDNA improving translation 4–6‐fold over the WT GLA sequence. We demonstrated that liver‐directed AAV8‐hGLA gene therapy in juvenile Fabry mice resulted in marked increase of α‐Gal A enzyme production and activity in the liver, with high amounts of enzyme secreted into the circulation. The secreted α‐Gal A was taken up by target organs, probably by receptor‐mediated endocytosis [63, 64], reducing lyso‐Gb3 accumulation to WT levels at relatively low AAV vector doses. The therapeutic effect showed an evident dose‐dependent increase in the α‐Gal A enzyme activity levels, with all the doses reaching supraphysiological levels of activity. The higher efficiency of the codon‐optimized GLA cDNA, compared with the WT GLA cDNA version, was evident in quantitative and semi‐quantitative studies performed, such as determination of protein levels (Western blot), enzyme activity, and lyso‐Gb3 accumulation, both in blood and target organs. Since the phenotype in Fabry mice is mild due to slow progression of the disease [39], this difference was not evident in the histological analysis since the sensitivity to detect differences in lyso‐Gb3 accumulation by immunostaining is lower than in biochemical methods. In the functional hot plate test for thermal nociception, correction by both vectors was similar due to their high efficiency to clear lyso‐Gb3 from tissues.

Various liver‐specific AAV‐mediated gene therapy studies for FD have been tested in mice, with all of them requiring higher AAV vector doses to obtain complete or sub‐optimal Gb3 or lyso‐Gb3 clearance in target tissues [25, 28, 32, 33, 65, 66, 67, 68, 69, 70], when compared to the lower doses used in this study that resulted in the complete or almost complete lyso‐Gb3 normalization in blood and tissues. However, minor discrepancies may be present due to differences in the titer assay standardization when comparing AAV doses from different labs. Importantly, some of these reports include pre‐clinical studies performed in support of clinical trials. For example, an experiment conducted by Sangamo therapeutics in Fabry mice showed ~70% reduction of lyso‐Gb3 in the kidneys and ~80% in the liver and plasma when treated with an AAV2/6 vector containing a codon‐optimized *GLA* cDNA at 2.0E12 vg/kg, and only 40% clearance in the plasma and tissues with a dose of 2.5E11 vg/kg [70]. This drug (ST‐920) has been tested in a Phase I/II clinical trial at a dose of 2.63E13 vg/kg resulting in improved disease severity score compared with ERT [71], and has been granted the fast‐track designation by the US FDA for the phase 3 trials in patients with Fabry disease. A liver‐directed AAV gene therapy is also being evaluated by Freeline Therapeutics. In the mouse studies done with the AAV8 serotype, a dose of 2.0E12 vg/kg of the clinical vector (FLT‐190) resulted in normalization of lyso‐Gb3 in plasma, heart and liver, but the reduction of lyso‐Gb3 in kidney reached only 65% [28]. Another clinical trial is being conducted by 4D Molecular Therapeutics using an ubiquitous gene promoter (CAG), where mouse studies showed normalization of lyso‐Gb3 in plasma, heart and liver when mice were dosed with 1.0E13 vg/kg of their clinical vector (4D‐310), while only 75% reduction was observed in kidney at this AAV dose [31]. Recently, a study sponsored by UniQure showed almost complete and complete clearance of lyso‐Gb3 in kidney and heart of Fabry mice with doses of 3.3E14 and 7.7E15 vg/kg, respectively, of a AAV5‐GLA vector driven by a liver‐specific promoter [68]. When comparing these existing therapies with the results obtained in the present study, a proof of the advanced performance is clearly established. We have shown that the lowest tested dose of 3.0E11vg/kg AAV8 pSMD2 h*GLA*_CO2 vector reduced 95% substrate in the liver, 87% in the kidneys, and 85% in the heart, and 100% correction was obtained with the 3.0E12 vg/kg AAV dose, with significantly better efficacy compared to ERT treatment (Figure 2B–E). We should take also consider that of ERT efficacy may be reduced since it was started at P90, rather than at P30 as the AAV treatment.

Additionally, the steady levels of α‐Gal A enzyme activity and lyso‐Gb3 observed in both the short‐ and long‐term experiments, without immunosuppression, suggest that the treatment does not generate significant levels of neutralizing antibodies against the liver‐produced human enzyme and are in line with previous results showing the induction of immune tolerance after AAV‐mediated hepatic expression of α‐Gal A [33] and other therapeutic proteins [35, 72, 73], although the validation of this assumption requires experimental testing.

These data are indicative of the potential of the vector design and the codon‐optimized construct tested here. Developing a more potent vector will allow the use of lower vector doses to achieve the same therapeutic efficacy, potentially minimizing anti‐AAV capsid immune responses and genotoxicity, which are associated with administration of higher vector doses [51]. Although not directly assessed in our study, we also expect that our treatment will elicit an anti‐AAV humoral response, potentially limiting vector re‐administration.

It is interesting to note that plasma α‐Gal A levels in mice treated at the highest dose of AAV8 pSMD2_h*GLA* CO2 vectors experienced a drop in the activity level during the first month post treatment, which later stabilized until the termination of the experiment (Figure 2B). As a hypothesis, this reduction could be explained by the death of the hepatocytes having higher VCN due to ER stress, triggered by the high load of α‐Gal A enzyme production from the CO2 *GLA* cDNA. Consequently, the overall α‐Gal A levels were reduced in the mice transduced with the highest dose of h*GLA*_CO2 (3.0E13vg/kg) when compared with those transduced with the h*GLA* WT‐AAV vector. Therefore, this observation should be taken into consideration in the design of a clinical trial, although the dose able to completely normalize lyso‐Gb3 accumulation in plasma and tissues of Fabry mice was at least 10‐fold lower than this critical one. Another potential explanation could be the misfolding of the codon optimized variant, possibly resulting from altered translation kinetics. This may activate the unfolded protein response (UPR), thereby reducing therapeutic efficacy, particularly at very high AAV doses where higher protein production levels are present. However, other studies may be required to fully determine the causes of this drop in α‐Gal A production at very high AAV doses, as other alternative mechanisms may also be present.

The long‐term studies showed durability and efficacy of the gene therapy for over 11 months after vector dosing, with normalization of lyso‐Gb3 levels in plasma and target tissues, showing normal histological architecture. The therapeutic effect in the neurological phenotype was assessed by the hot plate test [32]. This test, which measures the nociceptive response to a heat stimulus, showed the complete protection of AAV‐treated Fabry mice, with latency times as WT untreated mice, suggesting that the proposed therapy can also address the pathology in the dorsal root ganglia and improve the animals' thermal nociception, providing strong support to the high efficiency of the treatment.

In Fabry disease, the first symptoms of the classical form often appear in childhood or early adolescence, whereas late‐onset FD is usually diagnosed in adulthood. This delayed diagnosis, combined with the low proliferation rate of hepatocytes during adolescence and adulthood, supports the hypothesis that liver‐directed gene therapy using AAV vectors—engineered to express *GLA* under the control of a liver‐specific promoter—may be an effective strategy for these patients. On the contrary, early diagnosis of the classical form, facilitated by the post‐natal screening programs already implemented in some countries, may benefit from permanent genome editing approaches which could prevent the loss of AAV DNA during hepatocyte proliferation in early life [36, 74, 75].

In conclusion, our work demonstrates that the AAV8‐GLA‐CO2 vector represents a promising novel therapy for treating juvenile and adult patients with FD, including those with the classical form who are diagnosed late or currently receiving enzyme replacement therapy. This vector was able to successfully rescue the pathological phenotype of a mouse model of FD, including neuropathic involvement, when it was injected in the blood circulation of juvenile presymptomatic animals, with an 84%–95% reduction in lyso‐Gb3 accumulation in target tissues with a dose as low as 3.0E11 vg/kg.

## Author Contributions

H.S., A.D., and A.F.M. contributed to conception and design, supervision, and results interpretation, and writing (original draft and review‐editing) of the manuscript. A.F.M. contributed to funding acquisition. R.D., G.R., J.B., M.F., D.F., A.V., A.I., G.B., and L.Z. contributed to investigation, analysis, and interpretation of data. All authors contributed to writing (review and editing) of the manuscript.

## Funding

This work was supported by the Telethon Foundation, Italy (Grant N. GGP20128) to A.F.M. The authors confirm independence from the sponsors; the content of the article has not been influenced by the sponsors.

## Ethics Statement

The experiments performed here were approved by the ICGEB OPBA (“Organismo Preposto al Benessere degli Animali”: Animal Welfare Committee) and by the Italian Ministry of Health (Authorization N. 365/2021‐PR).

## Consent

The authors have nothing to report.

## Conflicts of Interest

A.F.M. and H.S. are owners of a patent describing the codon optimized cDNA sequence for human alpha galactosidase (PCT/EP2024/055793). The remaining authors declare no conflicts of interest.

## Supporting information


**Table S1:** Characteristics of the different codon optimized GLA cDNAs. The table shows the similarity with the WT GLA cDNA, % of GC content, codon optimization index (CAI), tRNA adaptation index (tAI), Effective Number of Codons (ENC).
**Table S2:** Lyso‐Gb3 levels in plasma samples of the experiment shown in Figure 2. The table shows the relative values of lyso‐Gb3 (considering the GLA KO as 100%, expressed as Mean ± SD) of the experiment shown in Figure 2E.
**Table S3:** a‐Gla A enzyme activity of the liver samples of the experiment shown in Figure 4. The table shows the enzyme activity (expressed as mmole/mg/h) of the experiment shown in Figure 4B.
**Figure S1:** In vitro test of codon optimized GLA cDNAs. (A) Scheme of the codon‐optimized GLA constructs. The human GLA 201 transcript was used as a template to generate four codon‐optimized variants of the gene using online tools and manual alterations to the codon composition. These cDNAs were cloned into the pSMD2‐hAAT‐ApoE plasmid containing a liver‐specific promoter, and transfected into Huh‐7 liver cells for protein and enzyme activity assessments; (B) The graph shows the codon adaptation index (CAI) for each of the GLA cDNA variants; (C) The putative GpC islands are shown: (D) The Western blot assay of Huh‐7 transfected cells was done for cell extracts and supernatants (tissue culture medium) using anti‐a‐Gal A–specific antibody and normalized with the eGFP transfection control; (E) The bar graph shows the quantification of the blot shown in Panel D; (F) The bar graph shows the a‐Gal A enzyme activity in the cell extracts and in the cell culture medium.
**Figure S2:** cDNA sequences of the WT and codon optimized variants of the human GLA cDNA
**Figure S3:** Alignment of WT and codon optimized 3 (CO3) sequences of the human GLA cDNA. The figure shows the alignment of the cDNA and amino acid sequences performed with online tools (vectorbuilder.com).
**Figure S4:** In vivo assessment of codon optimized cDNAs. Western blot of plasma of the WT mice of Figure 1, which were transduced with the different AAV‐GLA cDNAs. Each lane corresponds to a single animals. The same amount of protein of samples of the mice transduced with the WT GLA cDNA were loaded in all gels to allow the comparison across the different blots.
**Figure S5:** In vivo assessment of codon optimized cDNAs. Western blot of liver protein extracts of the mice of Figure 1, which were transduced with the different AAV‐GLA cDNAs. Each lane corresponds to a single animals. The same amount of protein of samples of the mice transduced with the WT GLA cDNA were loaded in all gels to allow the comparison across the different blots.
**Figure S6:** Characterization of Fabry KO mice. Male Fabry KO animals aged 2‐, 3‐, 4‐, 5‐, and 6 months were sacrificed, the liver, kidney, and heart were harvested, and blood was collected to extract plasma. Proteins were extracted from the tissue homogenates and a‐GalA enzyme activity assay was done in (A) liver (B) kidneys and (C) heart for all the animals (nmoles/mg/h). (D) a‐GalA enzyme activity was analyzed in the plasma isolated from the collected blood (nmoles/ml/h). (E) Lyso‐Gb3 accumulation was measured in plasma of 5‐months old male hemizygous and wild‐type mice (ng/mL) with mass‐spectrometric analysis. (F) Kidney and liver sections of 5‐month‐old hemizygous and wild‐type male mice were cryopreserved for immunofluorescence with Anti‐Gb3 antibody (red), Phalloidin (green) and the nucleus was stained with Hoechst (blue). The white arrows mark the Gb3 accumulation in the hemizygous animals.
**Figure S7:** Assessment of the codon optimized cDNA variants in vivo in Fabry mice treated with AAV based gene therapy (Figure 2). (A and B) All the animals were weighed at intermediate time points throughout the experiment along with untreated controls; (C) Proteins extracted from liver homogenates were used to run an SDS‐PAGE gel. Western blot analysis was done with the treated animals with Anti‐a‐Gal A antibody to detect a‐Gal A proteins on the blot and HSP70 specific antibody was used as a housekeeping protein. The blot shown is representative of all the treatment groups. (D) The bar graph represents the quantified and normalized values of the bands obtained from all the animals treated at different doses. (E) Quantitative RT‐PCR was done with genomic DNA extracted from liver homogenates to amplify the promoter region on the AAV8 pSMD2 vector to estimate viral genome copies/cell in the treated animals. The red bars and dotted bars indicate the mean of vgp/cell present in the livers of AAV8 pSMD2_hGLA WT and AAV8 pSMD2_hGLA CO2 treated mice at different doses.

## Data Availability

The data that support the findings of this study are available from the corresponding author upon reasonable request.

## References

[1] R. O. Brady , A. E. Gal , R. M. Bradley , E. Martensson , A. L. Warshaw , and L. Laster , “Enzymatic Defect in Fabry's Disease,” New England Journal of Medicine 276 (1967): 1163–1167. 6023233 10.1056/NEJM196705252762101

[2] A. Ortiz , D. P. Germain , R. J. Desnick , et al., “Fabry Disease Revisited: Management and Treatment Recommendations for Adult Patients,” Molecular Genetics and Metabolism 123 (2018): 416–427. 29530533 10.1016/j.ymgme.2018.02.014

[3] Y. A. Zarate and R. J. Hopkin , “Fabry's Disease,” Lancet 372 (2008): 1427–1435. 18940466 10.1016/S0140-6736(08)61589-5

[4] M. Arends , C. E. Hollak , and M. Biegstraaten , “Quality of Life in Patients With Fabry Disease: A Systematic Review of the Literature,” Orphanet Journal of Rare Diseases 10 (2015): 77. 26076709 10.1186/s13023-015-0296-8 PMC4501376

[5] D. P. Germain , “Fabry Disease,” Orphanet Journal of Rare Diseases 5 (2010): 30. 21092187 10.1186/1750-1172-5-30 PMC3009617

[6] A. Linhart , C. Kampmann , J. L. Zamorano , et al., “Cardiac Manifestations of Anderson‐Fabry Disease: Results From the International Fabry Outcome Survey,” European Heart Journal 28 (2007): 1228–1235. 17483538 10.1093/eurheartj/ehm153

[7] R. J. Desnick , R. Brady , J. Barranger , et al., “Fabry Disease, an Under‐Recognized Multisystemic Disorder: Expert Recommendations for Diagnosis, Management, and Enzyme Replacement Therapy,” Annals of Internal Medicine 138 (2003): 338–346. 12585833 10.7326/0003-4819-138-4-200302180-00014

[8] P. J. Meikle , J. J. Hopwood , A. E. Clague , and W. F. Carey , “Prevalence of Lysosomal Storage Disorders,” JAMA 281 (1999): 249–254. 9918480 10.1001/jama.281.3.249

[9] H. Y. Lin , K. W. Chong , J. H. Hsu , et al., “High Incidence of the Cardiac Variant of Fabry Disease Revealed by Newborn Screening in the Taiwan Chinese Population,” Circulation. Cardiovascular Genetics 2 (2009): 450–456. 20031620 10.1161/CIRCGENETICS.109.862920

[10] V. Gragnaniello , C. Cazzorla , D. Gueraldi , et al., “Light and Shadows in Newborn Screening for Lysosomal Storage Disorders: Eight Years of Experience in Northeast Italy,” International Journal of Neonatal Screening 10 (2023): 1–13. 38248631 10.3390/ijns10010003 PMC10801488

[11] M. Spada , S. Pagliardini , M. Yasuda , et al., “High Incidence of Later‐Onset Fabry Disease Revealed by Newborn Screening,” American Journal of Human Genetics 79 (2006): 31–40. 16773563 10.1086/504601 PMC1474133

[12] R. J. Hopkin , J. L. Jefferies , D. A. Laney , et al., “The Management and Treatment of Children With Fabry Disease: A United States‐Based Perspective,” Molecular Genetics and Metabolism 117 (2016): 104–113. 26546059 10.1016/j.ymgme.2015.10.007

[13] J. T. Clarke , M. L. West , J. Bultas , and R. Schiffmann , “The Pharmacology of Multiple Regimens of Agalsidase Alfa Enzyme Replacement Therapy for Fabry Disease,” Genetics in Medicine: Official Journal of the American College of Medical Genetics 9 (2007): 504–509. 17700388 10.1097/GIM.0b013e318133fb1b

[14] C. M. Eng , M. Banikazemi , R. E. Gordon , et al., “A Phase 1/2 Clinical Trial of Enzyme Replacement in Fabry Disease: Pharmacokinetic, Substrate Clearance, and Safety Studies,” American Journal of Human Genetics 68 (2001): 711–722. 11179018 10.1086/318809 PMC1274483

[15] Y. A. Ioannou , K. M. Zeidner , R. E. Gordon , and R. J. Desnick , “Fabry Disease: Preclinical Studies Demonstrate the Effectiveness of α‐Galactosidase A Replacement in Enzyme‐Deficient Mice,” American Journal of Human Genetics 68 (2001): 14–25. 11115376 10.1086/316953 PMC1234907

[16] D. F. Moore , M. Ries , E. L. Forget , and R. Schiffmann , “Enzyme Replacement Therapy in Orphan and Ultra‐Orphan Diseases: The Limitations of Standard Economic Metrics as Exemplified by Fabry‐Anderson Disease,” PharmacoEconomics 25 (2007): 201–208. 17335306 10.2165/00019053-200725030-00003

[17] S. M. Rombach , C. E. Hollak , G. E. Linthorst , and M. G. Dijkgraaf , “Cost‐Effectiveness of Enzyme Replacement Therapy for Fabry Disease,” Orphanet Journal of Rare Diseases 8 (2013): 29. 23421808 10.1186/1750-1172-8-29 PMC3598841

[18] W. Mauhin , O. Lidove , D. Amelin , et al., “Deep Characterization of the Anti‐Drug Antibodies Developed in Fabry Disease Patients, a Prospective Analysis From the French Multicenter Cohort FFABRY,” Orphanet Journal of Rare Diseases 13 (2018): 127. 30064518 10.1186/s13023-018-0877-4 PMC6069887

[19] M. Holida , A. Linhart , A. Pisani , et al., “A Phase III, Open‐Label Clinical Trial Evaluating Pegunigalsidase Alfa Administered Every 4 Weeks in Adults With Fabry Disease Previously Treated With Other Enzyme Replacement Therapies,” Journal of Inherited Metabolic Disease 48 (2025): e12795. 39381863 10.1002/jimd.12795 PMC11667655

[20] M. Lenders , E. R. Menke , and E. Brand , “Progress and Challenges in the Treatment of Fabry Disease,” BioDrugs: Clinical Immunotherapeutics, Biopharmaceuticals and Gene Therapy 39 (2025): 517–535. 40310476 10.1007/s40259-025-00723-3 PMC12185606

[21] R. Schiffmann , O. Goker‐Alpan , M. Holida , et al., “Pegunigalsidase Alfa, a Novel PEGylated Enzyme Replacement Therapy for Fabry Disease, Provides Sustained Plasma Concentrations and Favorable Pharmacodynamics: A 1‐Year Phase 1/2 Clinical Trial,” Journal of Inherited Metabolic Disease 42 (2019): 534–544. 30834538 10.1002/jimd.12080

[22] A. Markham , “Migalastat: First Global Approval,” Drugs 76 (2016): 1147–1152. 27351440 10.1007/s40265-016-0607-y

[23] J. M. Domm , S. K. Wootton , J. A. Medin , and M. L. West , “Gene Therapy for Fabry Disease: Progress, Challenges, and Outlooks on Gene‐Editing,” Molecular Genetics and Metabolism 134 (2021): 117–131. 34340879 10.1016/j.ymgme.2021.07.006

[24] M. Umer and D. K. Kalra , “Treatment of Fabry Disease: Established and Emerging Therapies,” Pharmaceuticals (Basel) 16 (2023): 320. 37259462 10.3390/ph16020320 PMC9967779

[25] M. G. Biferi , M. Cohen‐Tannoudji , A. Garcia‐Silva , et al., “Systemic Treatment of Fabry Disease Using a Novel AAV9 Vector Expressing Alpha‐Galactosidase A,” Molecular Therapy ‐ Methods & Clinical Development 20 (2021): 1–17. 33335943 10.1016/j.omtm.2020.10.016 PMC7725667

[26] L. D'Antiga , U. Beuers , G. Ronzitti , et al., “Gene Therapy in Patients With the Crigler‐Najjar Syndrome,” New England Journal of Medicine 389 (2023): 620–631. 37585628 10.1056/NEJMoa2214084

[27] T. Higuchi , Y. Shimada , Y. Takahashi , F. Kato , T. Ohashi , and H. Kobayashi , “Restoration of Peripheral Neuropathy in Fabry Mice via Intrathecal Administration of an Adeno‐Associated Virus Vector Encoding mGLA cDNA,” Molecular Genetics and Metabolism 143 (2024): 108545. 39068683 10.1016/j.ymgme.2024.108545

[28] J. M. Jeyakumar , A. Kia , L. C. S. Tam , et al., “Preclinical Evaluation of FLT190, a Liver‐Directed AAV Gene Therapy for Fabry Disease,” Gene Therapy 30 (2023): 487–502. 36631545 10.1038/s41434-022-00381-y PMC10284695

[29] A. C. Nathwani , U. M. Reiss , E. G. Tuddenham , et al., “Long‐Term Safety and Efficacy of Factor IX Gene Therapy in Hemophilia B,” New England Journal of Medicine 371 (2014): 1994–2004. 25409372 10.1056/NEJMoa1407309 PMC4278802

[30] S. W. Pipe , F. W. G. Leebeek , M. Recht , et al., “Gene Therapy With Etranacogene Dezaparvovec for Hemophilia B,” New England Journal of Medicine 388 (2023): 706–718. 36812434 10.1056/NEJMoa2211644

[31] K. Whittlesey , G. Brooks , P. Szymanski , et al., “A Targeted AAV Gene Therapy Product Candidate, 4D‐310, for the Treatment of Fabry Disease: Intravenous Biodistribution, Transgene Expression and Safety in Non‐ Human Primates,” Molecular Therapy: The Journal of the American Society of Gene Therapy 29 (2021): 115.

[32] R. J. Ziegler , M. Cherry , C. M. Barbon , et al., “Correction of the Biochemical and Functional Deficits in Fabry Mice Following AAV8‐Mediated Hepatic Expression of Alpha‐Galactosidase A,” Molecular Therapy: The Journal of the American Society of Gene Therapy 15 (2007): 492–500. 10.1038/sj.mt.6300066 17191071

[33] R. J. Ziegler , S. M. Lonning , D. Armentano , et al., “AAV2 Vector Harboring a Liver‐Restricted Promoter Facilitates Sustained Expression of Therapeutic Levels of Alpha‐Galactosidase A and the Induction of Immune Tolerance in Fabry Mice,” Molecular Therapy: The Journal of the American Society of Gene Therapy 9 (2004): 231–240. 14759807 10.1016/j.ymthe.2003.11.015

[34] A. M. Keeler , W. Zhan , S. Ram , K. A. Fitzgerald , and G. Gao , “The Curious Case of AAV Immunology,” Molecular Therapy: The Journal of the American Society of Gene Therapy 33 (2025): 1946–1965. 40156190 10.1016/j.ymthe.2025.03.037 PMC12126790

[35] F. Mingozzi , Y. L. Liu , E. Dobrzynski , et al., “Induction of Immune Tolerance to Coagulation Factor IX Antigen by In Vivo Hepatic Gene Transfer,” Journal of Clinical Investigation 111 (2003): 1347–1356. 12727926 10.1172/JCI16887 PMC154443

[36] G. Bortolussi , L. Zentillin , J. Vanikova , et al., “Life‐Long Correction of Hyperbilirubinemia With a Neonatal Liver‐Specific AAV‐Mediated Gene Transfer in a Lethal Mouse Model of Crigler‐Najjar Syndrome,” Human Gene Therapy 25 (2014): 844–855. 25072305 10.1089/hum.2013.233 PMC4175423

[37] F. Collaud , G. Bortolussi , L. Guianvarc'h , et al., “Preclinical Development of an AAV8‐hUGT1A1 Vector for the Treatment of Crigler‐Najjar Syndrome,” Molecular Therapy ‐ Methods & Clinical Development 12 (2019): 157–174. 30705921 10.1016/j.omtm.2018.12.011 PMC6348934

[38] G. Ronzitti , G. Bortolussi , R. van Dijk , et al., “A Translationally Optimized AAV‐UGT1A1 Vector Drives Safe and Long‐Lasting Correction of Crigler‐Najjar Syndrome,” Molecular Therapy Methods & Clinical Development 3 (2016): 16049. 27722180 10.1038/mtm.2016.49 PMC5052023

[39] T. Ohshima , G. J. Murray , W. D. Swaim , et al., “Alpha‐Galactosidase A Deficient Mice: A Model of Fabry Disease,” Proceedings of the National Academy of Sciences of the United States of America 94 (1997): 2540–2544. 9122231 10.1073/pnas.94.6.2540 PMC20124

[40] U. Gioia , S. Tavella , P. Martinez‐Orellana , et al., “SARS‐CoV‐2 Infection Induces DNA Damage, Through CHK1 Degradation and Impaired 53BP1 Recruitment, and Cellular Senescence,” Nature Cell Biology 25 (2023): 550–564. 36894671 10.1038/s41556-023-01096-x PMC10104783

[41] G. Bortolussi , A. Iaconcig , G. Canarutto , et al., “CRISPR‐Cas9‐Mediated Somatic Correction of a One‐Base Deletion in the Ugt1a Gene Ameliorates Hyperbilirubinemia in Crigler‐Najjar Syndrome Mice,” Molecular Therapy Methods & Clinical Development 31 (2023): 101161. 38094199 10.1016/j.omtm.2023.101161 PMC10716028

[42] D. S. Bangari , K. M. Ashe , R. J. Desnick , et al., “α‐Galactosidase A Knockout Mice,” American Journal of Pathology 185 (2015): 651–665. 25553976 10.1016/j.ajpath.2014.11.004

[43] M. F. Macedo , R. Quinta , C. S. Pereira , and M. C. Sa Miranda , “Enzyme Replacement Therapy Partially Prevents Invariant Natural Killer T Cell Deficiency in the Fabry Disease Mouse Model,” Molecular Genetics and Metabolism 106 (2012): 83–91. 22425450 10.1016/j.ymgme.2012.02.014

[44] R. Quinta , D. Rodrigues , M. Assuncao , et al., “Reduced Glucosylceramide in the Mouse Model of Fabry Disease: Correction by Successful Enzyme Replacement Therapy,” Gene 536 (2014): 97–104. 24334116 10.1016/j.gene.2013.11.073

[45] K. M. Ashe , E. Budman , D. S. Bangari , et al., “Efficacy of Enzyme and Substrate Reduction Therapy With a Novel Antagonist of Glucosylceramide Synthase for Fabry Disease,” Molecular Medicine 21 (2015): 389–399. 25938659 10.2119/molmed.2015.00088 PMC4559530

[46] S. Jabbarzadeh‐Tabrizi , M. Boutin , T. S. Day , et al., “Assessing the Role of Glycosphingolipids in the Phenotype Severity of Fabry Disease Mouse Model,” Journal of Lipid Research 61 (2020): 1410–1423. 32868283 10.1194/jlr.RA120000909 PMC7604726

[47] L. A. George , S. K. Sullivan , A. Giermasz , et al., “Hemophilia B Gene Therapy With a High‐Specific‐Activity Factor IX Variant,” New England Journal of Medicine 377 (2017): 2215–2227. 29211678 10.1056/NEJMoa1708538 PMC6029626

[48] S. Rangarajan , L. Walsh , W. Lester , et al., “AAV5‐Factor VIII Gene Transfer in Severe Hemophilia A,” New England Journal of Medicine 377 (2017): 2519–2530. 29224506 10.1056/NEJMoa1708483

[49] V. Muczynski and A. C. Nathwani , “AAV Mediated Gene Therapy for Haemophilia B: From the Early Attempts to Modern Trials,” Thrombosis Research 236 (2024): 242–249. 38383218 10.1016/j.thromres.2020.12.033

[50] F. Puzzo and M. A. Kay , “The deLIVERed Promises of Gene Therapy: Past, Present, and Future of Liver‐Directed Gene Therapy,” Molecular Therapy: The Journal of the American Society of Gene Therapy 33 (2025): 1966–1987. 40156191 10.1016/j.ymthe.2025.03.041 PMC12126789

[51] P. Colella , G. Ronzitti , and F. Mingozzi , “Emerging Issues in AAV‐Mediated In Vivo Gene Therapy,” Molecular Therapy Methods & Clinical Development 8 (2018): 87–104. 29326962 10.1016/j.omtm.2017.11.007 PMC5758940

[52] P. Heinke , F. Rost , J. Rode , et al., “Diploid Hepatocytes Drive Physiological Liver Renewal in Adult Humans,” Cell Systems 13 (2022): 499–507. 35649419 10.1016/j.cels.2022.05.001

[53] Y. Magami , T. Azuma , H. Inokuchi , et al., “Cell Proliferation and Renewal of Normal Hepatocytes and Bile Duct Cells in Adult Mouse Liver,” Liver 22 (2002): 419–425. 12390477 10.1034/j.1600-0676.2002.01702.x

[54] F. Mingozzi and K. A. High , “Overcoming the Host Immune Response to Adeno‐Associated Virus Gene Delivery Vectors: The Race Between Clearance, Tolerance, Neutralization, and Escape,” Annual Review of Virology 4 (2017): 511–534. 10.1146/annurev-virology-101416-041936 28961410

[55] B. Bertin , P. Veron , C. Leborgne , et al., “Capsid‐Specific Removal of Circulating Antibodies to Adeno‐Associated Virus Vectors,” Scientific Reports 10 (2020): 864. 31965041 10.1038/s41598-020-57893-z PMC6972890

[56] L. G. Chicoine , C. L. Montgomery , W. G. Bremer , et al., “Plasmapheresis Eliminates the Negative Impact of AAV Antibodies on Microdystrophin Gene Expression Following Vascular Delivery,” Molecular Therapy: The Journal of the American Society of Gene Therapy 22 (2014): 338–347. 24196577 10.1038/mt.2013.244 PMC3916040

[57] Z. C. Elmore , D. K. Oh , K. E. Simon , M. M. Fanous , and A. Asokan , “Rescuing AAV Gene Transfer From Neutralizing Antibodies With an IgG‐Degrading Enzyme,” JCI Insight 5 (2020): 1–11. 10.1172/jci.insight.139881 PMC7566709 32941184

[58] P. O. Ilyinskii , C. Roy , A. Michaud , et al., “Readministration of High‐Dose Adeno‐Associated Virus Gene Therapy Vectors Enabled by ImmTOR Nanoparticles Combined With B Cell‐Targeted Agents,” PNAS Nexus 2 (2023): pgad394. 38024395 10.1093/pnasnexus/pgad394 PMC10673641

[59] P. O. Ilyinskii , C. J. Roy , J. LePrevost , G. L. Rizzo , and T. K. Kishimoto , “Enhancement of the Tolerogenic Phenotype in the Liver by ImmTOR Nanoparticles,” Frontiers in Immunology 12 (2021): 637469. 34113339 10.3389/fimmu.2021.637469 PMC8186318

[60] C. Leborgne , E. Barbon , J. M. Alexander , et al., “IgG‐Cleaving Endopeptidase Enables In Vivo Gene Therapy in the Presence of Anti‐AAV Neutralizing Antibodies,” Nature Medicine 26 (2020): 1096–1101. 10.1038/s41591-020-0911-7 32483358

[61] A. Meliani , F. Boisgerault , R. Hardet , et al., “Antigen‐Selective Modulation of AAV Immunogenicity With Tolerogenic Rapamycin Nanoparticles Enables Successful Vector Re‐Administration,” Nature Communications 9 (2018): 4098. 10.1038/s41467-018-06621-3 PMC6173722 30291246

[62] V. Monteilhet , S. Saheb , S. Boutin , et al., “A 10 Patient Case Report on the Impact of Plasmapheresis Upon Neutralizing Factors Against Adeno‐Associated Virus (AAV) Types 1, 2, 6, and 8,” Molecular Therapy: The Journal of the American Society of Gene Therapy 19 (2011): 2084–2091. 21629225 10.1038/mt.2011.108 PMC3222518

[63] M. M. Ivanova , J. Dao , N. Kasaci , B. Adewale , J. Fikry , and O. Goker‐Alpan , “Rapid Clathrin‐Mediated Uptake of Recombinant Alpha‐Gal‐A to Lysosome Activates Autophagy,” Biomolecules 10 (2020): 1–20. 10.3390/biom10060837 PMC7356514 32486191

[64] T. Prabakaran , R. Nielsen , J. V. Larsen , et al., “Receptor‐Mediated Endocytosis of Alpha‐Galactosidase A in Human Podocytes in Fabry Disease,” PLoS One 6 (2011): e25065. 21949853 10.1371/journal.pone.0025065 PMC3176300

[65] N. Boukharov , S. Yuan , W. Ruangsirluk , et al., “Developing Gene Therapy for Mitigating Multisystemic Pathology in Fabry Disease: Proof of Concept in an Aggravated Mouse Model,” Human Gene Therapy 35 (2024): 680–694. 38970423 10.1089/hum.2023.222

[66] Y. Hayashi , Y. Sehara , R. Watano , et al., “Therapeutic Strategy for Fabry Disease by Intravenous Administration of Adeno‐Associated Virus 2 or 9 in Alpha‐Galactosidase A‐Deficient Mice,” Journal of Gene Medicine 25 (2023): e3560. 37392007 10.1002/jgm.3560

[67] S. C. Jung , I. P. Han , A. Limaye , et al., “Adeno‐Associated Viral Vector‐Mediated Gene Transfer Results in Long‐Term Enzymatic and Functional Correction in Multiple Organs of Fabry Mice,” Proceedings of the National Academy of Sciences of the United States of America 98 (2001): 2676–2681. 11226298 10.1073/pnas.051634498 PMC30197

[68] J. M. P. Liefhebber , G. Brasser , E. A. Spronck , et al., “Preclinical Efficacy and Safety of Adeno‐Associated Virus 5 Alpha‐Galactosidase: A Gene Therapy for Fabry Disease,” Molecular Therapy Methods & Clinical Development 32 (2024): 101375. 39687734 10.1016/j.omtm.2024.101375 PMC11646755

[69] K. Ogawa , Y. Hirai , M. Ishizaki , et al., “Long‐Term Inhibition of Glycosphingolipid Accumulation in Fabry Model Mice by a Single Systemic Injection of AAV1 Vector in the Neonatal Period,” Molecular Genetics and Metabolism 96 (2009): 91–96. 19091614 10.1016/j.ymgme.2008.10.017

[70] M. Yasuda , M. W. Huston , S. Pagant , et al., “AAV2/6 Gene Therapy in a Murine Model of Fabry Disease Results in Supraphysiological Enzyme Activity and Effective Substrate Reduction,” Molecular Therapy ‐ Methods & Clinical Development 18 (2020): 607–619. 32775495 10.1016/j.omtm.2020.07.002 PMC7396970

[71] Sangamo_Therapeutics , A Phase I/II, Multicenter, Open‐Label, Single‐Dose, Dose‐Ranging Study to Assess the Safety and Tolerability of ST‐920, an AAV2/6 Human Alpha Galactosidase A Gene Therapy, in Subjects With Fabry Disease (STAAR) (clinicaltrials.Gov). In Editor eds. Book A Phase I/II, Multicenter, Open‐Label, Single‐Dose, Dose‐Ranging Study to Assess the Safety and Tolerability of ST‐920, an AAV2/6 Human Alpha Galactosidase A Gene Therapy, in Subjects With Fabry Disease (STAAR) (clinicaltrials.Gov), 2024.

[72] S. O. Han , G. Ronzitti , B. Arnson , et al., “Low‐Dose Liver‐Targeted Gene Therapy for Pompe Disease Enhances Therapeutic Efficacy of ERT via Immune Tolerance Induction,” Molecular Therapy Methods & Clinical Development 4 (2017): 126–136. 28344998 10.1016/j.omtm.2016.12.010 PMC5363303

[73] G. D. Keeler , D. M. Markusic , and B. E. Hoffman , “Liver Induced Transgene Tolerance With AAV Vectors,” Cellular Immunology 342 (2019): 103728. 29576315 10.1016/j.cellimm.2017.12.002 PMC5988960

[74] S. C. Cunningham , A. P. Dane , A. Spinoulas , G. J. Logan , and I. E. Alexander , “Gene Delivery to the Juvenile Mouse Liver Using AAV2/8 Vectors,” Molecular Therapy: The Journal of the American Society of Gene Therapy 16 (2008): 1081–1088. 10.1038/mt.2008.72 18414478

[75] L. Wang , H. Wang , P. Bell , D. McMenamin , and J. M. Wilson , “Hepatic Gene Transfer in Neonatal Mice by Adeno‐Associated Virus Serotype 8 Vector,” Human Gene Therapy 23 (2012): 533–539. 22098408 10.1089/hum.2011.183 PMC3360497

