# Supplementary material for: Liver Gene Therapy in Fabry Disease Mice With Low Doses of rAAV2/8 Expressing a Codon‐Optimized h GLA cDNA Results in Long‐Term Disease Correction
Source: J Inherit Metab Dis. 2026 Apr 27;49:e70188. doi: 10.1002/jimd.70188 (PMC13112331; doi:10.1002/jimd.70188)
Supplement: Supplementary file 1 — Table S1: Characteristics of the different codon optimized GLA cDNAs. The table shows the similarity with the WT GLA cDNA, % of GC content, codon optimization index (CAI), tRNA adaptation index (tAI), Effective Number of Codons (ENC). Table S2: Lyso‐Gb3 levels in plasma samples of the experiment shown in Figure 2. The table shows the relative values of lyso‐Gb3 (considering the GLA KO as 100%, expressed as Mean ± SD) of the experiment shown in Figure 2E. Table S3: a‐Gla A enzyme activity of the liver samples of the experiment shown in Figure 4. The table shows the enzyme activity (expressed as mmole/mg/h) of the experiment shown in Figure 4B. Figure S1: In vitro test of codon optimized GLA cDNAs. (A) Scheme of the codon‐optimized GLA constructs. The human GLA 201 transcript was used as a template to generate four codon‐optimized variants of the gene using online tools and manual alterations to the codon composition. These cDNAs were cloned into the pSMD2‐hAAT‐ApoE plasmid containing a liver‐specific promoter, and transfected into Huh‐7 liver cells for protein and enzyme activity assessments; (B) The graph shows the codon adaptation index (CAI) for each of the GLA cDNA variants; (C) The putative GpC islands are shown: (D) The Western blot assay of Huh‐7 transfected cells was done for cell extracts and supernatants (tissue culture medium) using anti‐a‐Gal A–specific antibody and normalized with the eGFP transfection control; (E) The bar graph shows the quantification of the blot shown in Panel D; (F) The bar graph shows the a‐Gal A enzyme activity in the cell extracts and in the cell culture medium. Figure S2: cDNA sequences of the WT and codon optimized variants of the human GLA cDNA Figure S3: Alignment of WT and codon optimized 3 (CO3) sequences of the human GLA cDNA. The figure shows the alignment of the cDNA and amino acid sequences performed with online tools (vectorbuilder.com). Figure S4: In vivo assessment of codon optimized cDNAs. Western blot of plasma of t [file JIMD-49-0-s001.pdf]

Supplementary Material

Liver gene therapy in Fabry disease mice with low doses of rAAV2/8 expressing a codon-optimised h*GLA* cDNA results in long-term disease correction

Himanshi Saxena[1], Rossana Domenis[2], Giulia Romano[1], Jessica Biasizzo[2], Martina Ferro[3], Dania Ferino[2], Antonio Vicidomini[1], Alessandra Iaconcig[1], Giulia Bortolussi[1], Lorena Zentilin[1], Andrea Dardis[3], and Andrés F. Muro[1]

[1] International Centre for Genetic Engineering and Biotechnology, Padriciano, 99, 34149-Trieste, Italy.

[2] Institute of Clinical Pathology, Department of Laboratory Medicine, University Hospital of Udine, P. Le Santa Maria Della Misericordia 15, 33100-Udine, Italy.

[3] Regional Coordinator Centre for Rare Diseases, University Hospital of Udine, P. Le Santa Maria Della Misericordia 15, 33100-Udine, Italy.

Supplementary Materials and Methods

rAAV vector production

The AAV vectors used in this study are based on AAV type 2 backbone, and infectious vectors were prepared by the AAV Vector Unit at ICGEB Trieste (https://www.icgeb.org/avu-core-facility.html) in HEK293 cells by a cross-packing approach whereby the vector was packaged into AAV capsid 8, as described previously (Bortolussi et al 2014).

Preparation of total protein extracts and Western Blot analysis

Whole livers were harvested, reduced to powder using a mortar pestle with liquid nitrogen, and stored at -80°C. Around 20-25mg of liver powder was homogenized using a mechanical homogenizer (IKA ULTRA-TURRAX T25), in 100 µl protein lysis buffer. The homogenate was centrifuged at 13,000 rcf for 15 minutes at 4°C. Supernatants were transferred into a fresh tube and stored at -80°C. Bradford (Bio-Rad) method was used to determine total protein concentration. Coomassie Brilliant Blue G-250 (200µl) was added to 1 µl of protein lysate in a transparent 96-well plate. Bovine Serum Albumin (BSA) at different concentrations were used as standard samples. Using a multi-plate reader (Perkin Elmer Envision Plate Reader, Waltham, MA), the absorption of the samples and standards was measured at 595 nm. The protein concentration was determined by plotting the absorbance obtained against the BSA standards reference curve. Different lysis buffers were used depending on the analytical purposes. Lysis buffer for the purpose of western blot analysis was composed of 50 mM Hepes (pH 7.4), 150 mM Sodium chloride (NaCl), 1 mM EDTA,

0.5% NP40, Protease inhibitor, and water, whereas the buffer used to extract proteins to perform α-Gal A enzymatic assay consists of 28 mM citric acid, 44 mM Disodium phosphate, 1% TritonX-100 and protease inhibitor.

Proteins extracted from liver homogenates of treated and untreated mice (15 µg) or plasma diluted 1:50 were used to perform an SDS-PAGE. The samples were denatured in 1x protein loading buffer (250 mM Tris-HCl pH 6.8, 10% SDS, 0.5% Bromophenol blue, 50% Glycerol, 500 mM DTT) and loaded onto a 4-12% precasted gel.

The proteins were transferred to a Polyvinylidene fluoride (PVDF) membrane using a Lightning Blot™ System (Perkin Elmer). The membrane was blocked in blocking buffer (5% milk in 0.1% PBS-Tween 20) for 2-4 hours followed by incubation in primary antibody (rabbit anti-h-α-Gal A, Sino Biologicals, 1:3000, cat: 12078-R001; rat anti-HSP70 Enzo Life Science, 1:3000, cat ADI-SPA-815-D) diluted in the blocking buffer overnight at 4°C on a shaking surface. The primary antibody was detected with a goat anti-rabbit IgG horseradish peroxidase (IgG-HRP) or anti-rat, respectively (rat anti-IgG-HRP, Bethyl Laboratories, 1:3000, cat A110-305P; rabbit anti-IgG-HRP, Dako, 1:3000, cat P0448). Finally, the membrane was developed with Enhanced Chemiluminescence (ECL – ThermoFisher Scientific) to be imaged using a ChemiDoc imaging system (Biorad). The band intensities on the images were measured using Image Lab software 6.0.1 (Biorad) for a quantitative evaluation of the western blot analysis.

Histology

The right lobe of the liver and/or one kidney were harvested during the sacrifice of the treated and untreated mice in 4% PFA (4% paraformaldehyde in PBS). The tissues were fixed in the solution at 4°C for 24 hours. The buffer was changed to 20% sucrose solution (20% sucrose, 0.02% sodium azide, in PBS) post-incubation.

For immunofluorescence the tissues were frozen at optimal cutting temperature compound and sliced into 4 µm slices on histological slides. The slides with tissue sections were stored at -20°C until further use. Similarly, in the case of Huh-7 cells, the medium was removed from the well plate, and cells were washed with PBS. 4% PFA was added to the cells and incubated for 20 minutes at room temperature followed by PBS wash. Slides of liver and kidney sections were subjected to antigen retrieval (10 mM Sodium citrate buffer pH 6.0) by baking the slides in the microwave for 40 minutes. The slides were washed with PBS and blocked with blocking buffer (5% BSA in 0.4% PBS-Triton X 100) for 2 hours at room temperature. Next, sections were incubated with the primary antibody in 2.5% BSA in 0.4% PBS-triton X 100 for 2 hours at room temperature (mouse anti-Gb3, Amsbio, 1:250, cat AMS.A2506; Alexa fluor 488 anti Phalloidin, Invitrogen, 1:500, act A12379). Incubation with a secondary antibody (Alexa Fluor 568 mouse anti-IgG, Invitrogen, 1:500, cat A11004) was performed in wash buffer for 2 hours at room temperature and then counterstained with Hoechst and mounted in Mowiol. Slides were imaged with Leica fluorescent microscope (Leica DMIL led) coupled with DFC450C camera. Images were processed with ImageJ software.

For immunohistochemistry (IHC) the liver left lobe was fixed in 10% formalin overnight at 4°C, washed, and cryoprotected in 20% sucrose/0.02% sodium azide at 4°C before paraffin embedding and sectioning at 4 µm. Histological staining involved deparaffinization and rehydration (Bio-Clear and Unyhol – Bio-Optica), blocking of

endogenous HRP and AP (ReadyProbes™, Invitrogen) 15 min at RT, antigen retrieval (10 mM citrate pH 6, 95°C, 30 min), and blocking with 10% normal goat serum (Dako, 30 min). Primary antibodies against Gb3 (mouse monoclonal, 1:200 -Amsio) and hα-Gal A (rabbit monoclonal, 1:200 -Sino Biological) were incubated for 1 hour at room temperature. Duplex staining was performed with the Abcam M&R IHC kit (HRP/Green & AP/Red) per manufacturer's protocol. Nuclei were counterstained with Gill's hematoxylin N°3 (Bio-Optica), and sections were mounted in Eukitt (Sigma-Aldrich). Images were captured using a Leica ICC50 W camera on a DM750 Leica microscope.

α-Gal A enzyme activity

α-Gal A enzyme activity test was done with Huh-7 cell extract and supernatant medium, and murine plasma and tissue including liver, kidney, and heart as a phenotypic marker of Fabry disease as previously described with minor modifications (Smith et al 2014; Unzueta et al 2015). Protein extracted from the cells and tissues using citrate lysis buffer indicated in the protein extraction section was used as samples, along with crude supernatant and plasma diluted according to the requirement with PBS. Initially, 4MU (4-Methylumbelliferone, Cat# M1381 Sigma Aldrich) was used to prepare stocks of different concentrations (2, 4, 10, 20, 40, 80, and 100 pmoles) to be used as standards to generate a reference curve. In a black opaque 96-well plate, a 2 µl enzyme-containing sample (protein from cell/tissue, supernatant medium, or blood plasma) was added. To this, 20 µl of 4MUG (4-Methyl-umbelliferyl-alpha-D-galactopyranoside, Cat#M7633 Sigma Aldrich) (2.46 mM 4MUG in 0.1 M Citrate/ 0.2 M phosphate buffer pH 4.5) substrate was added and mixed gently by pipetting. The plate was incubated at 37°C for the reaction to take place. After 1

hour of incubation, 200 µl of stop solution (200 mM glycine-NaOH Buffer pH 10.4) was added to stop the reaction. Previously prepared standards were also added to the plate diluted in 200 µl of stop solution. The released fluorescence from the samples and the standards was measured at excitation 365 nm and emission 450 nm wavelengths with a multi-plate reader (Perkin Elmer Envision Plate Reader, Waltham, MA). For the analysis, the RFU obtained was normalized with the appropriate blank samples, and 4MU units (B) released by the samples were calculated using the standard curve and expressed in nmoles/ml/hr or nmoles/mg/hr as indicated in the figures.

Supplementary References

Bortolussi G, Baj G, Vodret S, Viviani G, Bittolo T, Muro AF (2014) Age-dependent pattern of cerebellar susceptibility to bilirubin neurotoxicity in vivo in mice. *Disease models & mechanisms* 7: 1057-1068.

Smith W, Jantti J, Oja M, Saloheimo M (2014) Comparison of intracellular and secretion-based strategies for production of human alpha-galactosidase A in the filamentous fungus Trichoderma reesei. *BMC Biotechnol* 14: 91.

Unzueta U, Vazquez F, Accardi G, et al (2015) Strategies for the production of difficult-to-express full-length eukaryotic proteins using microbial cell factories: production of human alpha-galactosidase A. *Appl Microbiol Biotechnol* 99: 5863-5874.

Supplementary Tables and Figures

Table S1. Characteristics of the different codon optimised *GLA* cDNAs. The table shows the similarity with the WT *GLA* cDNA, % of GC content, codon optimisation index (CAI), tRNA adaptation index (tAI), Effective Number of Codons (ENC).

Sequence Name	Similarity (% vs. WT)	GC content (%)*	CAI**	tAI**	ENC****
h***GLA***_WT	100	49.0	0.74	0.34	56.2
h***GLA***_CO1	76.5	51.4	0.73	0.35	55.6
h***GLA***_CO2	78.1	66.0	0.98	0.41	21.2
h***GLA***_CO3	80.2	57.0	0.89	0.39	38.7
h***GLA***_CO4	80.8	50.2	0.80	0.36	45.7

Table S2. Lyso-Gb3 levels in plasma samples of the experiment shown in Figure 2. The table shows the relative values of lyso-Gb3 (considering the GLA KO as 100%, expressed as Mean±SD) of the experiment shown in Figure 2E.

Dose/Treatment	AAV8-h*GLA* WT	AAV8-h*GLA* CO02	NO gene therapy
Gla KO 3.0E13 vg/kg	0.22±0.04	1.25±1.02	
Gla KO 1.0E13 vg/kg	0.96±0.36	0.32±0.12	
Gla KO 3.0E12 vg/kg	6.56±3.86	1.87±1.04	
Gla KO 3.0E11 vg/kg	38.57±24.91	10.46±6.48	
C57Bl/6 WT			0.29±0.03
Gla KO			100.00±1.61
Gla KO ERT			2.36±2.71

Table S3. a-Gla A enzyme activity of the liver samples of the experiment shown in Figure 4. The table shows the enzyme activity (expressed as mmole/mg/hr) of the esperiment shown in Figure 4B.

Days	WT mean	CO2 mean	α-Gal A ko Untreated
60	7425.5	11221.1	0.5
90	6453.2	8094.4	0.5
120	8264.2	9819.7	0.6
150	8760.5	10846.7	0.5
180	9795.0	10558.0	0.7
210	8476.2	9271.9	0.4
240	8763.2	9879.8	0.4
270	3625.0	8780.4	0.4
300	6983.1	7133.3	0.4
330	7133.3	8468.9	0.6
360	4170.9	4171.5	0.6

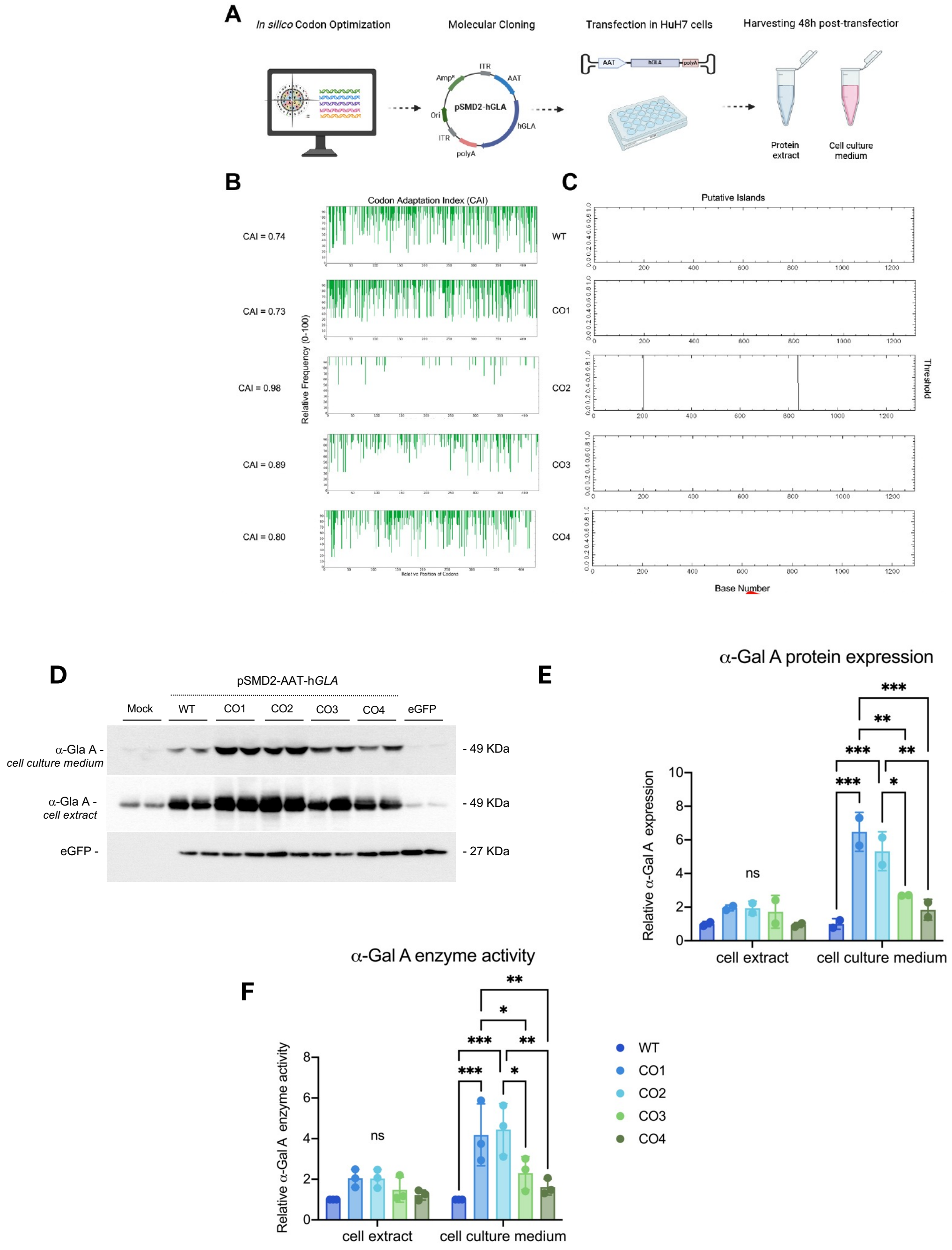

Figure S1. ***In vitro*** **test of codon optimised GLA cDNAs. A)** Scheme of the codon-optimised GLA constructs. The human GLA 201 transcript was used as a template to generate four codon-optimised variants of the gene using online tools and manual alterations to the codon composition. These cDNAs were cloned into the pSMD2-hAAT-ApoE plasmid containin a liver-specific promorer, and transfected into Huh-7 liver cellsfor protein and enzyme activity assessments; **B)** The graph shows the codon adaptation index (CAI) for each of the GLA cDNA variants; **C)** The putative GpC islands are shown: **D)** The Western blot assay of Huh-7 transfected cells was done for cell extracts and supernatants (tissue culture medium) using *anti-α-Gal A* –specific antibody and normalised with the eGFP transfection control; **E)** The bar graph shows the quantification of the blot shown in Panel D; **F)** The bar graph shows the *α-Gal A* enzyme activity in the cell extracts and in the cell culture medium.

WILD TYPE (hGLA WT)

```
ATG CAG CTG AGG AAC CCA GAA CTA CAT CTG GGC TGC GCG CTT GCG CTT CGC TTC CTG GCC CTC
GTT TCC TGG GAC ATC CCT GGG GCT AGA GCA CTG GAC AAT GGA TTG GCA AGG ACG CCT ACC ATG
GGC TGG CTG CAC TGG GAG CGC TTC ATG TGC AAC CTT GAC TGC CAG GAA GAG CCA GAT TCC TGC
ATC AGT GAG AAG CTC TTC ATG GAG ATG GCA GAG CTC ATG GTC TCA GAA GGC TGG AAG GAT GCA
GGT TAT GAG TAC CTC TGC ATT GAT GAC TGT TGG ATG GCT CCC CAA AGA GAT TCA GAA GGC AGA
CTT CAG GCA GAC CCT CAG CGC TTT CCT CAT GGG ATT CGC CAG CTG GCT AAT TAT GTT CAC AGC
AAA GGA CTG AAG CTA GGG ATT TAT GCA GAT GTT GGA AAT AAA ACC TGC GCA GGC TTC CCT GGG
AGT TTT GGA TAC TAC GAC ATT GAT GCC CAG ACC TTT GCT GAC TGG GGA GTA GAT CTG CTA AAA
TTT GAT GGT TGT TAC TGT GAC AGT TTG GAA AAT TTG GCA GAT GGT TAT AAG CAC ATG TCC TTG
GCC CTG AAT AGG ACT GGC AGA AGC ATT GTG TAC TCC TGT GAG TGG CCT CTT TAT ATG TGG CCC
TTT CAA AAG CCC AAT TAT ACA GAA ATC CGA CAG TAC TGC AAT CAC TGG CGA AAT TTT GCT GAC
ATT GAT GAT TCC TGG AAA AGT ATA AAG AGT ATC TTG GAC TGG ACA TCT TTT AAC CAG GAG AGA
ATT GTT GAT GTT GCT GGA CCA GGG GGT TGG AAT GAC CCA GAT ATG TTA GTG ATT GGC AAC TTT
GGC CTC AGC TGG AAT CAG CAA GTA ACT CAG ATG GCC CTC TGG GCT ATC ATG GCT GCT CCT TTA
TTC ATG TCT AAT GAC CTC CGA CAC ATC AGC CCT CAA GCC AAA GCT CTC CTT CAG GAT AAG GAC
GTA ATT GCC ATC AAT CAG GAC CCC TTG GGC AAG CAA GGG TAC CAG CTT AGA CAG GGA GAC AAC
TTT GAA GTG TGG GAA CGA CCT CTC TCA GGC TTA GCC TGG GCT GTA GCT ATG ATA AAC CGG CAG
GAG ATT GGT GGA CCT CGC TCT TAT ACC ATC GCA GTT GCT TCC CTG GGT AAA GGA GTG GCC TGT
AAT CCT GCC TGC TTC ATC ACA CAG CTC CTC CCT GTG AAA AGG AAG CTA GGG TTC TAT GAA TGG
ACT TCA AGG TTA AGA AGT CAC ATA AAT CCC ACA GGC ACT GTT TTG CTT CAG CTA GAA AAT ACA
ATG CAG ATG TCA TTA AAA GAC TTA CTT TAA
```

hGLA CO1

```
ATG CAG CTC CGC AAC CCA GAG CTC CAT CTT GGG TGT GCT CTC GCT CTT CGA TTC CTT GCA CTG
GTC AGT TGG GAT ATC CCG GGA GCT AGA GCT TTG GAT AAC GGT CTC GCA CGC ACT CCC ACA ATG
GGA TGG CTT CAC TGG GAG CGA TTT ATG TGC AAC CTG GAC TGC CAG GAA GAG CCG GAT AGC TGT
ATA TCT GAG AAG CTT TTT ATG GAG ATG GCG GAA TTG ATG GTC AGT GAA GGC TGG AAA GAC GCG
GGC TAC GAA TAT CTC TGT ATC GAC GAT TGT TGG ATG GCA CCA CAA CGC GAT AGC GAA GGC AGG
CTC CAG GCT GAT CCA CAG AGG TTT CCC CAC GGA ATA CGA CAG CTG GCT AAC TAT GTG CAC AGC
AAG GGC CTC AAA CTG GGA ATC TAC GCT GAC GTG GGC AAT AAG ACG TGC GCC GGT TTC CCG GGG
TCT TTC GGT TAC TAC GAC ATT GAC GCC CAA ACT TTT GCT GAC TGG GGT GTG GAT CTT CTC AAG
TTT GAC GGC TGT TAC TGC GAC TCC CTC GAA AAT TTG GCT GAT GGT TAC AAG CAC ATG TCT CTT
GCC TTG AAT CGC ACC GGC CGC TCC ATC GTG TAC TCT TGC GAG TGG CCG TTG TAT ATG TGG CCC
TTT CAA AAA CCG AAC TAC ACA GAA ATA AGA CAG TAT TGC AAC CAC TGG AGA AAC TTC GCT GAT
ATC GAC GAT AGC TGG AAA TCT ATT AAA TCT ATT CTT GAT TGG ACG AGT TTT AAT CAA GAG CGA
ATT GTG GAC GTT GCG GGG CCG GGA GGG TGG AAC GAC CCC GAT ATG CTG GTT ATC GGA AAT TTT
GGC CTT TCC TGG AAT CAG CAG GTT ACC CAG ATG GCC CTG TGG GCT ATT ATG GCC GCT CCA CTC
TTC ATG AGC AAT GAT TTG CGC CAC ATC AGT CCA CAA GCG AAG GCT CTC TTG CAG GAT AAG GAT
GTG ATT GCT ATC AAC CAA GAT CCG CTG GGC AAG CAG GGG TAT CAG TTG AGA CAA GGA GAT AAC
TTC GAA GTT TGG GAG CGG CCC CTG AGT GGT TTG GCC TGG GCA GTG GCG ATG ATA AAT CGA CAA
GAA ATA GGA GGA CCC AGG AGT TAT ACT ATT GCT GTA GCA TCC CTT GGG AAA GGT GTC GCG TGT
AAC CCC GCT TGT TTT ATT ACA CAA CTG CTG CCT GTT AAG AGA AAA CTG GGC TTT TAC GAG TGG
ACC TCT CGG CTC AGA TCC CAC ATC AAC CCG ACA GGC ACC GTT CTT CTG CAA CTG GAG AAT ACG
ATG CAG ATG AGC CTC AAG GAC TTG TTG TAA
```

hGLA CO2

```
ATG CAG CTG CGC AAC CCC GAG CTG CAC CTG GGC TGC GCC CTG GCC CTG CGC TTC CTG GCC CTG
GTC AGC TGG GAC ATC CCC GGC GCC CGC GCC CTG GAC AAC GGC CTG GCC CGC ACC CCC ACC ATG
GGC TGG CTG CAC TGG GAG CGC TTC ATG TGC AAC CTG GAC TGC CAG GAG GAG CCC GAC AGC TGC
ATC AGC GAG AAG CTG TTT ATG GAG ATG GCC GAG CTG ATG GTC AGC GAG GGC TGG AAG GAC GCC
GGC TAC GAG TAC CTG TGC ATC GAC GAC TGC TGG ATG GCC CCC CAG CGC GAC AGC GAG GGC CGC
CTG CAG GCC GAC CCC CAG CGC TTC CCC CAC GGA ATC CGC CAG CTG GCC AAC TAC GTG CAC AGC
AAG GGC CTG AAG CTG GGC ATC TAC GCC GAC GTG GGC AAC AAG ACC TGC GCC GGC TTC CCC GGC
AGC TTC GGC TAC TAC GAC ATC GAC GCC CAG ACC TTC GCC GAC TGG GGC GTG GAC CTG CTG AAG
TTC GAC GGC TGC TAC TGC GAC AGC CTG GAG AAC CTG GCC GAC GGC TAC AAG CAC ATG AGC CTG
GCC CTG AAC CGC ACC GGC CGC AGC ATC GTG TAC AGC TGC GAG TGG CCC CTG TAT ATG TGG CCC
TTC CAG AAG CCC AAC TAC ACC GAG ATC CGC CAG TAC TGC AAC CAC TGG CGC AAC TTC GCC GAC
ATC GAC GAC AGC TGG AAG AGC ATC AAG AGC ATC CTG GAC TGG ACC AGC TTC AAC CAG GAG CGC
ATC GTG GAC GTG GCC GGC CCC GGC GGC TGG AAC GAC CCC GAC ATG CTG GTG ATC GGC AAC TTC
GGC CTG AGC TGG AAC CAG CAG GTG ACC CAG ATG GCC CTG TGG GCC ATT ATG GCC GCC CCC CTG
TTT ATG AGC AAC GAC CTG CGC CAC ATC AGC CCC CAG GCC AAG GCC CTG CTG CAG GAC AAG GAC
GTG ATC GCT ATC AAC CAG GAC CCC CTG GGC AAG CAG GGC TAC CAG CTG CGC CAG GGC GAC AAC
TTC GAG GTC TGG GAG CGC CCC CTG AGC GGC CTG GCC TGG GCC GTG GCT ATG ATC AAC CGC CAG
GAG ATC GGC GGC CCC CGC AGC TAC ACC ATC GCC GTG GCC AGC CTG GGC AAG GGC GTG GCC TGC
AAC CCC GCC TGC TTC ATC ACC CAG CTG CTG CCC GTG AAG CGC AAG CTG GGC TTC TAC GAG TGG
ACC AGC CGC CTG CGC AGC CAC ATC AAC CCC ACC GGC ACC GTG CTG CTG CAG CTG GAG AAC ACA
ATG CAG ATG AGC CTG AAG GAC CTG CTG TAA
```

hGLA CO3

```
ATG CAG TTG AGA AAC CCA GAG CTC CAC CTG GGC TGT GCC CTG GCA CTG AGG TTC CTG GCC CTT
GTG AGC TGG GAT ATC CCT GGG GCC AGG GCC TTG GAC AAC GGC TTG GCC CGC ACC CCC ACA ATG
GGC TGG CTG CAC TGG GAA CGC TTT ATG TGC AAT CTG GAC TGC CAG GAG GAG CCT GAC AGC TGT
ATC AGC GAG AAG CTC TTT ATG GAG ATG GCA GAG CTG ATG GTG TCT GAG GGA TGG AAG GAC GCC
GGC TAC GAA TAC CTG TGC ATT GAC GAT TGC TGG ATG GCT CCA CAG AGG GAC TCA GAA GGA CGC
CTG CAG GCT GAT CCC CAG AGA TTC CCC CAT GGA ATC CGC CAG CTG GCC AAC TAT GTG CAC AGC
AAA GGC CTG AAG CTG GGC ATC TAC GCC GAC GTG GGC AAC AAG ACC TGT GCT GGC TTC CCT GGC
TCC TTT GGA TAT TAC GAT ATC GAC GCT CAG ACC TTT GCT GAC TGG GGA GTG GAT CTC CTC AAG
TTT GAC GGC TGC TAC TGT GAC TCT CTG GAA AAC CTG GCA GAT GGC TAC AAG CAC ATG TCC CTG
GCT CTG AAC AGA ACA GGC CGC AGC ATT GTG TAC AGC TGC GAG TGG CCC CTG TAT ATG TGG CCC
TTC CAG AAG CCC AAC TAC ACA GAG ATC AGG CAG TAC TGC AAC CAC TGG AGG AAC TTT GCC GAC
ATT GAC GAC TCC TGG AAA TCT ATC AAG TCT ATC CTG GAT TGG ACA TCC TTC AAC CAA GAG CGG
ATC GTG GAC GTG GCT GGA CCT GGA GGC TGG AAT GAT CCA GAT ATG CTG GTG ATT GGA AAC TTC
GGG CTG TCT TGG AAC CAG CAG GTC ACT CAG ATG GCG CTG TGG GCC ATC ATG GCC GCC CCC CTC
TTT ATG AGC AAC GAC CTG CGC CAC ATT TCT CCT CAA GCC AAG GCC CTG CTC CAG GAC AAG GAC
GTC ATC GCC ATT AAT CAG GAT CCT CTG GGG AAG CAG GGC TAC CAG CTT AGA CAG GGA GAC AAT
TTT GAG GTG TGG GAG AGG CCT CTC TCT GGA CTT GCC TGG GCT GTG GCT ATG ATC AAC CGG CAG
GAA ATT GGT GGC CCC CGC TCC TAC ACC ATT GCT GTT GCC TCC TTG GGC AAG GGC GTG GCC TGT
AAC CCT GCC TGC TTC ATC ACC CAG CTC CTG CCT GTG AAG AGA AAA CTG GGA TTC TAC GAG TGG
ACC AGC CGG CTG CGG AGC CAC ATC AAT CCC ACC GGC ACC GTG CTG CTT CAG CTG GAG AAC ACC
ATG CAG ATG TCA CTG AAA GAT CTG CTG TGA
```

hGLA CO4

```
ATG CAG CTG AGA AAC CCT GAG CTG CAT CTG GGC TGT GCC CTG GCA CTA AGG TTC TTG GCT CTA
GTT  TCT TGG GAT ATC CCA GGA GCC AGA GCC CTG GAC AAC GGA TTG GCC AGG ACC CCA ACA ATG
GGC TGG CTC CAC TGG GAG AGG TTT ATG TGC AAC CTT GAC TGT CAG GAA GAG CCT GAC TCC TGC
ATC AGT GAA AAG CTC TTC ATG GAG ATG GCA GAA CTG ATG GTG TCT GAG GGC TGG AAG GAC GCT
GGC TAC GAG TAT CTG TGC ATT GAC GAC TGC TGG ATG GCC CCC CAG AGA GAC TCA GAG GGA AGG
TTG CAG GCT GAC CCA CAA AGA TTC CCC CAC GGA ATC AGA CAG CTG GCC AAC TAT GTC CAC AGC
AAA GGA CTC AAA CTG GGT ATA TAT GCA GAT GTA GGA AAC AAG ACC TGT GCT GGG TTT CCT GGT
AGC TTT GGT TAT TAT GAT ATA GAT GCT CAG ACC TTT GCT GAC TGG GGG GTG GAT CTG CTG AAA
TTT GAT GGA TGC TAC TGT GAC TCC TTA GAG AAT CTG GCT GAT GGT TAC AAG CAC ATG TCA CTG
GCT CTA AAC AGA ACT GGT AGA AGT ATT GTG TAC TCC TGC GAG TGG CCC TTG TAT ATG TGG CCT
TTC CAG AAA CCC AAC TAT ACA GAA ATC AGG CAG TAT TGC AAC CAC TGG AGG AAT TTT GCT GAT
ATA GAC GAC TCT TGG AAA AGT ATC AAA TCA ATC TTG GAT TGG ACA TCC TTC AAC CAA GAG AGG
ATT GTT GAT GTT GCT GGC CCA GGG GGT TGG AAT GAT CCT GAT ATG CTG GTG ATT GGA AAT TTT
GGG CTT AGC TGG AAC CAG CAA GTG ACC CAA ATG GCC TTA TGG GCT ATA ATG GCT GCA CCT CTG
TTT ATG AGC AAT GAC CTG AGG CAT ATC AGC CCT CAG GCA AAA GCC TTG CTG CAG GAT AAG GAT
GTG ATT GCA ATT AAC CAG GAC CCT CTT GGA AAG CAG GGT TAC CAG TTA AGA CAG GGA GAC AAT
TTT GAG GTG TGG GAA AGA CCT TTG TCT GGG CTG GCT TGG GCA GTG GCT ATG ATA AAC AGA CAG
GAG ATT GGT GGA CCC AGA TCC TAT ACC ATT GCT GTG GCT TCA CTG GGC AAG GGT GTA GCC TGC
AAT CCA GCT TGT TTT ATC ACC CAA CTG CTG CCT GTT AAG AGG AAG CTG GGC TTT TAT GAG TGG
ACC AGC AGG CTG AGA TCC CAC ATC AAC CCC ACT GGC ACT GTG TTG CTG CAG CTT GAG AAC ACA
ATG CAG ATG AGC CTG AAG GAC CTG CTG TGA
```

Figure S2. cDNA sequences of the WT and codon optimised variants of the human *GLA* cDNA

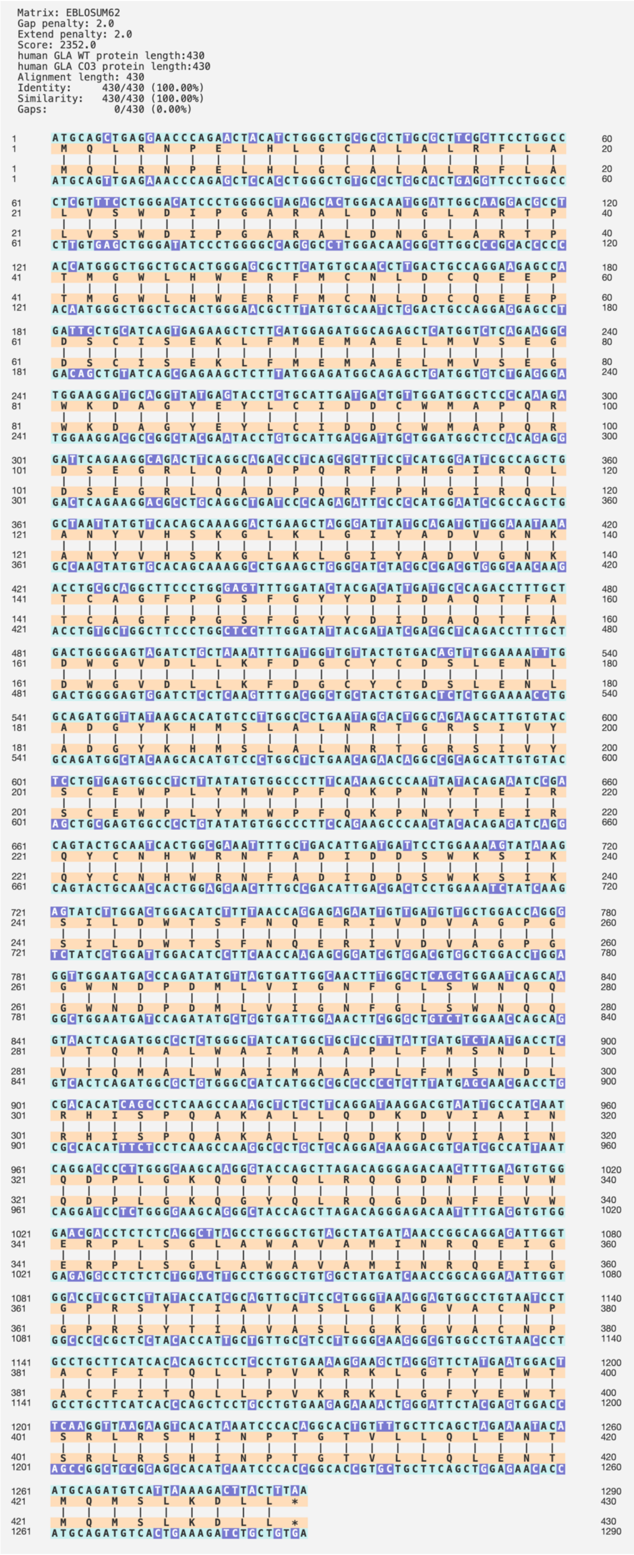

Figure S3. Alignment of of WT and codon optimised 3 (CO3) sequences of the human *GLA* cDNA. The figure shows the alignement of the cDNA and aminoacid sequences performed with online tools (vectorbuilder.com).

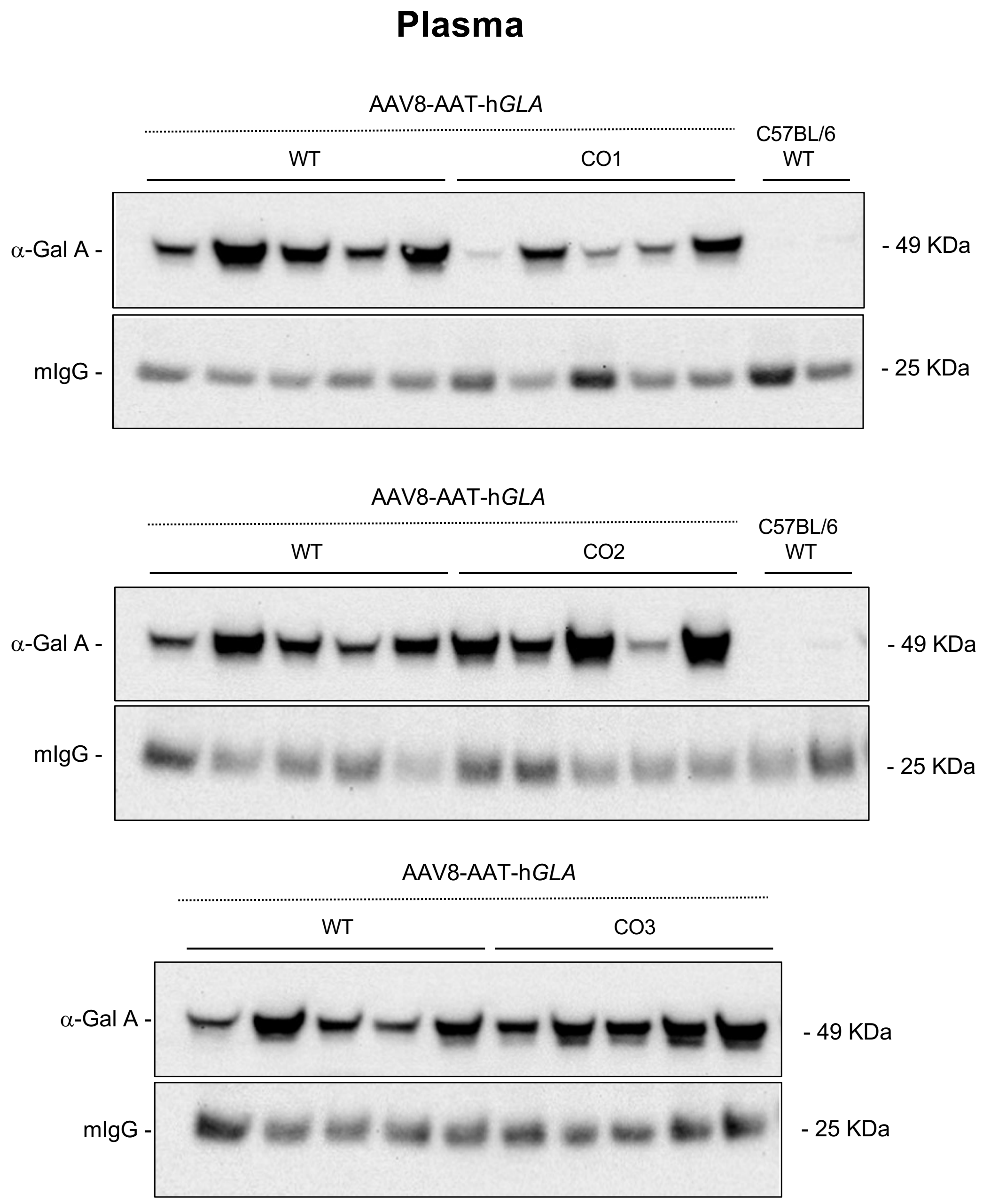

Figure S4. *In vivo* assessment of codon optimised cDNAs. Western blot of plasma of the WT mice of Figure 1, wich were transduced with the different AAV-GLA cDNAs. Each lane corresponds to a single animals. The same amount of protein of samples of the mice transduced with the WT GLA cDNA were loaded in all gels to allow the comparison across the different blots.

Liver

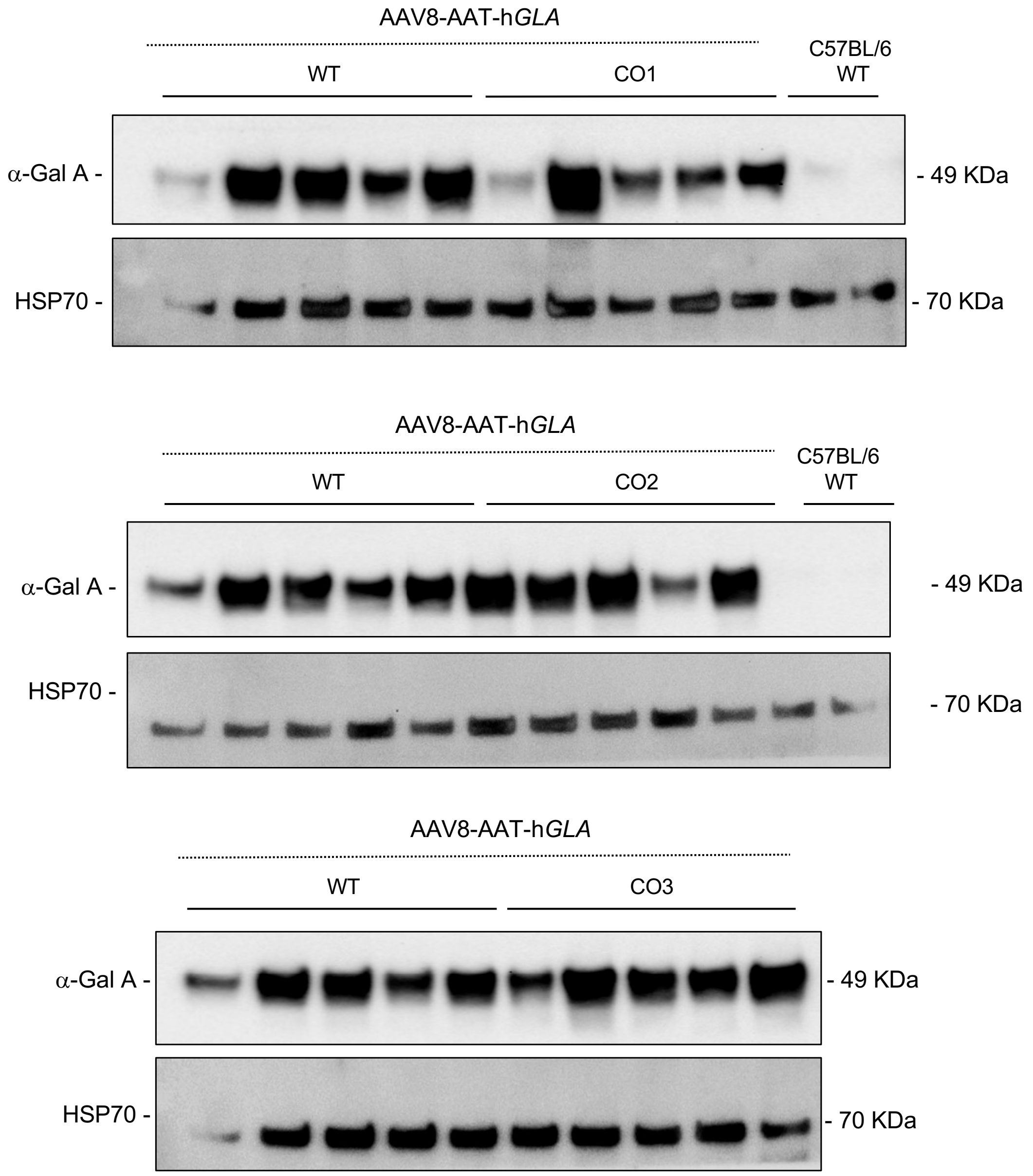

Figure S5. *In vivo* assessment of codon optimised cDNAs. Western blot of liver protein extracts of the mice of Figure 1, wich were transduced with the different AAV-*GLA* cDNAs. Each lane corresponds to a single animals. The same amount of protein of samples of the mice transduced with the WT *GLA* cDNA were loaded in all gels to allow the comparison across the different blots.

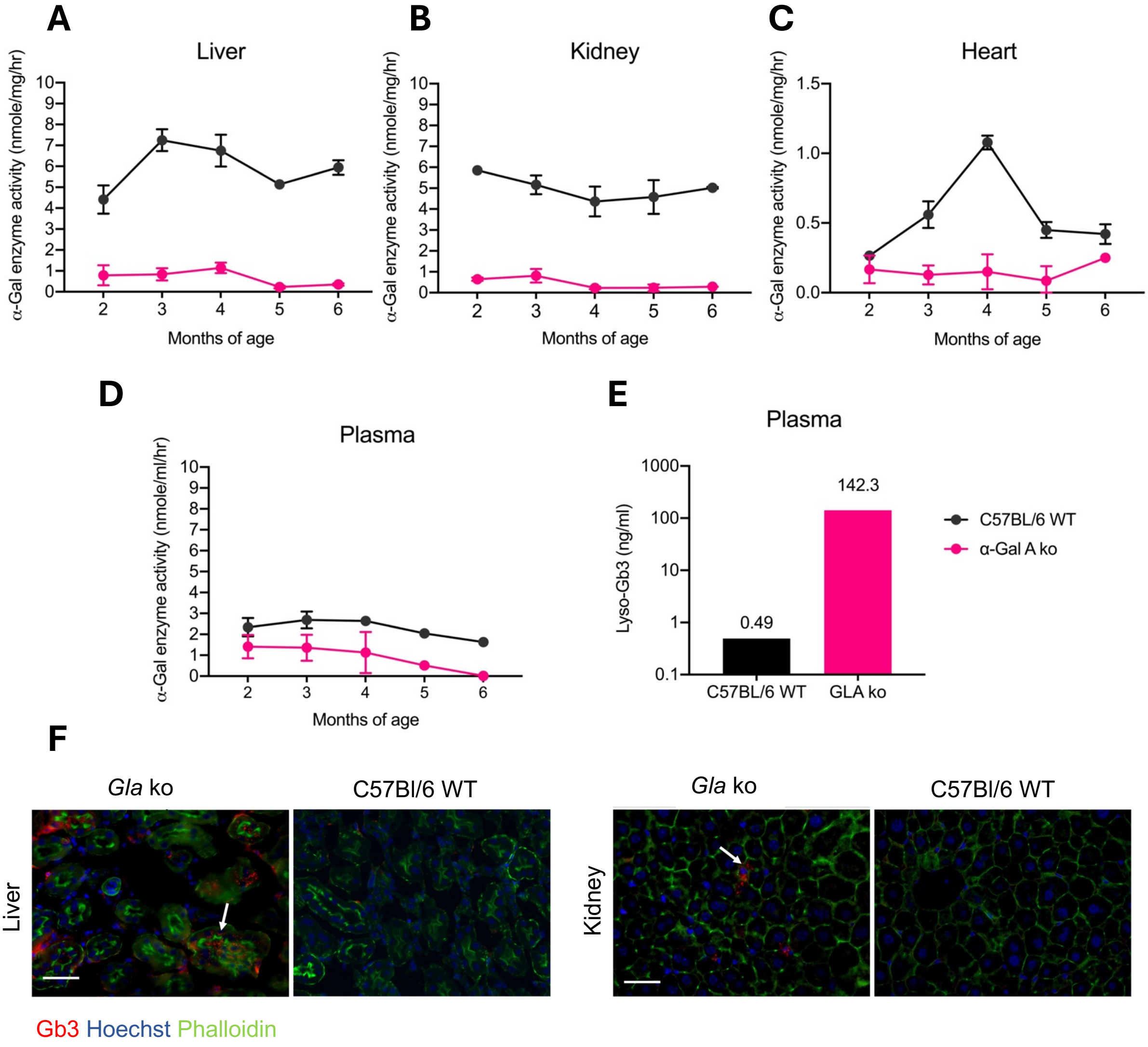

Figure S6. Characterisation of Fabry KO mice. Male Fabry KO animals aged 2-, 3-, 4-, 5-, and 6 months were sacrificed, the liver, kidney, and heart were harvested, and blood was collected to extract plasma. Proteins were extracted from the tissue homogenates and α-Gal A enzyme activity assay was done in **A)** liver **B)** kidneys and **C)** heart for all the animals (nmoles/mg/hr). **D)** α-Gal A enzyme activity was analysed in the plasma isolated from the collected blood (nmoles/ml/hr). **E)** Lyso-Gb3 accumulation was measured in plasma of 5-months old male hemizygous and wild-type mice (ng/ml) with mass-spectrometric analysis. **F)** Kidney and liver sections of 5-month-old hemizygous and wild-type male mice were cryopreserved for immunofluorescence with Anti-Gb3 antibody (red), Phalloidin (green) and the nucleus was stained with Hoechst (blue). The white arrows mark the Gb3 accumulation in the hemizygous animals.

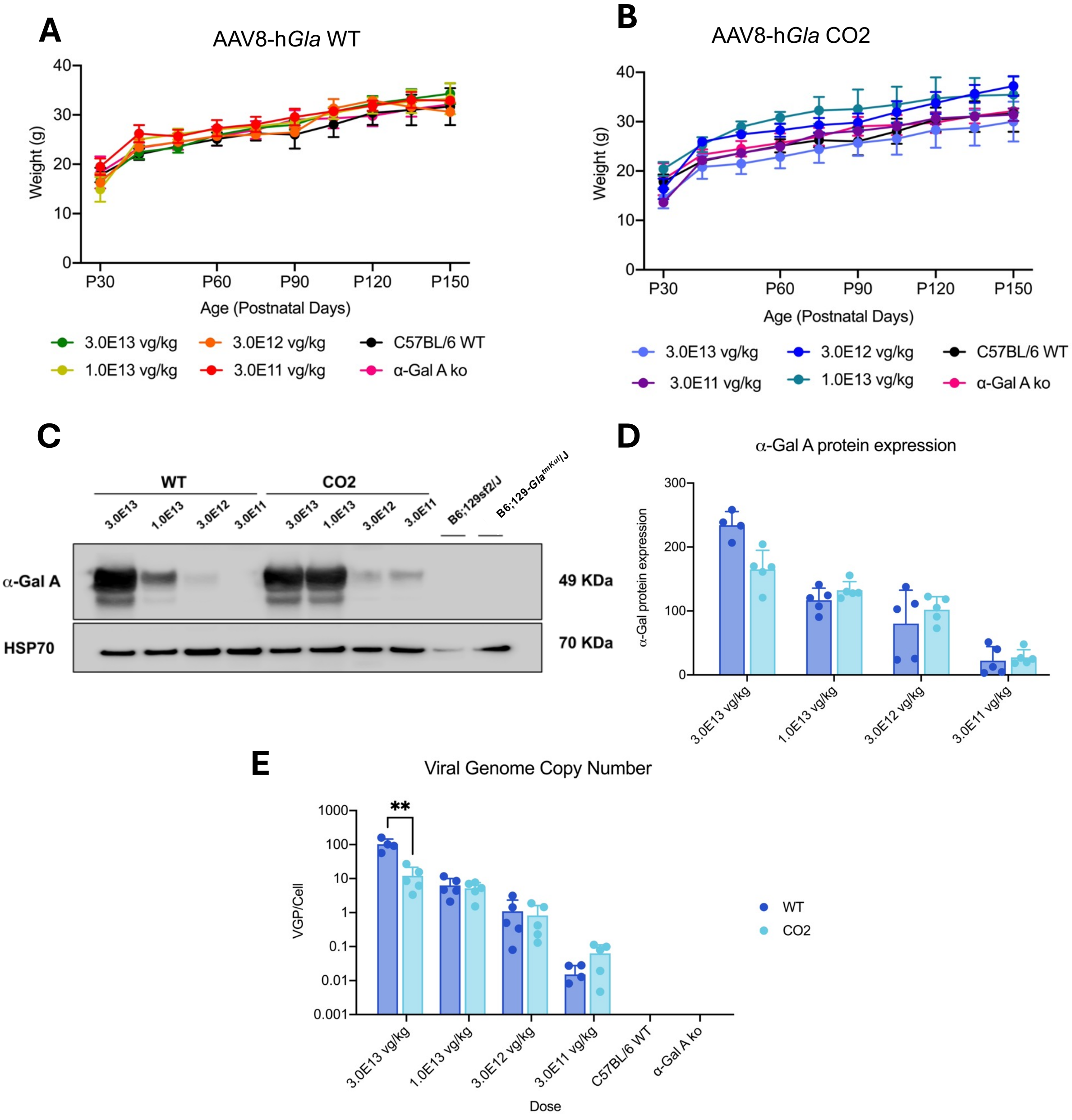

Figure S7. Assessment of the codon optimised cDNA variants *in vivo* in Fabry mice treated with AAV-based gene therapy (Figure 2). A and B) All the animals were weighed at intermediate time points throughout the experiment along with untreated controls; **C)** Proteins extracted from liver homogenates were used to run an SDS-PAGE gel. Western blot analysis was done with the treated animals with Anti-α-Gal A antibody to detect α-Gal A proteins on the blot and HSP70 specific antibody was used as a housekeeping protein. The blot shown is representative of all the treatment groups. **D)** The bar graph represents the quantified and normalized values of the bands obtained from all the animals treated at different doses. **E)** Quantitative RT-PCR was done with genomic DNA extracted from liver homogenates to amplify the promoter region on the AAV8 pSMD2 vector to estimate viral genome copies/cell in the treated animals. The red bars and dotted bars indicate the mean of vgp/cell present in the livers of AAV8 pSMD2_h*GLA* WT and AAV8 pSMD2_h*GLA* CO2 treated mice at different doses.
